# Maximum Softly Penalized Likelihood in Factor Analysis

**DOI:** 10.1017/psy.2026.10092

**Published:** 2026-02-18

**Authors:** Philipp Sterzinger, Ioannis Kosmidis, Irini Moustaki

**Affiliations:** 1 Department of Statistics, London School of Economics and Political Science, United Kingdom; 2 Department of Statistics, University of Warwick, United Kingdom

**Keywords:** Heywood cases, infinite estimates, singular variance components

## Abstract

Estimation in exploratory factor analysis often yields estimates on the boundary of the parameter space. Such occurrences, called Heywood cases, are characterized by non-positive variance estimates and can cause numerical instability, convergence failures, and misleading inferences. We derive sufficient conditions on the model and a penalty to the log-likelihood function that guarantee the existence of maximum penalized likelihood estimates in the interior of the parameter space, and that the corresponding estimators possess desirable asymptotic properties expected by the maximum likelihood estimator, namely, consistency and asymptotic normality. Consistency and asymptotic normality follow when penalization is soft enough, in a way that adapts to the information accumulation about the model parameters. We formally show, for the first time, that the penalties of Akaike (1987, *Psychometrika*, 52, 317–332) and Hirose et al. (2011, *Journal of Data Science*, 9, 243–259) to the log-likelihood of the normal linear factor model satisfy the conditions for existence, and, hence, deal with Heywood cases. Their vanilla versions, though, can result in questionable finite-sample properties in estimation, inference, and model selection. Our maximum softly-penalized likelihood (MSPL) framework ensures that the resulting estimation and inference procedures are asymptotically optimal. Through comprehensive simulation studies and real data analyses, we illustrate the desirable finite-sample properties of the MSPL estimators.

## 1 Introduction

Exploratory factor analysis has been widely used in social sciences and beyond to measure unobserved constructs of interest, such as ability, attitudes, and behaviors, and for dimensionality reduction. It has been noted early on in the factor analysis literature, particularly with the development of the more precise computational frameworks for maximum likelihood (ML) estimation in Jöreskog (1967) and Jöreskog and Lawley (1968), that the estimation of factor analysis models often results in improper solutions. Such improper solutions involve zero or negative estimates for error variances, and often correlation estimates greater than one in absolute value. Such occurrences are typically referred to as Heywood cases (Heywood, 1931). Martin and McDonald (1975) distinguish between an exact Heywood case in which at least one of the estimates of the error variances is zero but none are negative, and an ultra-Heywood case, in which at least one estimate of the error variances is negative. A zero error variance implies that there is no measurement error, and the factors fully explain the observed variable. That is rare in real applications but, at the same time, does not pose as much concern as negative estimates for error variances do. Causes of Heywood cases that have been reported in the literature are model and data dependent and include outliers, non-convergence of associated optimization procedures, under-identification, model misspecification, missing data, and sampling fluctuations combined with a true value close to the boundary for the parameter, small sample sizes, poorly defined factors, and factor over-extraction (see, e.g., Chen et al., 2001; Cooperman & Waller, 2022; Dillon et al., 1987; Kano, 1998; van Driel, 1978; and the references therein). Cooperman and Waller (2022) provide an up-to-date review of the causes, effects, and solutions to Heywood cases in confirmatory and exploratory factor analysis.

The presence of Heywood cases in factor analysis has practical implications. It can produce parameter estimates, standard errors, factor scores, and goodness-of-fit test statistics that cannot be trusted. Cooperman and Waller (2022) found, through a simulation study, that Heywood cases increase the standard errors of factor loadings and bias the factor scores upward. Eliminating items that correspond to estimates which display a Heywood case often moves the Heywood problem to one of the remaining items.

An approach to handle Heywood cases, especially when they are suspected to be due to sampling fluctuations, is by restricting the estimates of the error variances to 0,∞) either explicitly or by setting negative estimates to zero (see Gerbing & Anderson, 1987 for a discussion). However, this violates regularity conditions of ML estimation, leading to estimators and testing procedures with properties that are hard to evaluate. Another common approach is to impose priors on the loadings, error variances, or both to avoid improper solutions. Estimation, then, proceeds either using a likelihood-based approach with the prior information incorporated via a penalty term (see, e.g., Akaike, 1987; Hirose et al., 2011; Lee, 1981; Martin & McDonald, 1975) or by posterior sampling through MCMC (see, e.g., Lee & Song, 2002). For example, Martin and McDonald (1975) proposed a Bayesian estimation framework in which they maximize not the likelihood but the posterior density, using a prior distribution for the error covariance matrix that assigns zero probability to negative values. They assume a prior distribution that is almost uniform, except that it decreases to zero at the point where an error variance is equal to zero. Lee (1981) also investigated the form of the posterior density under different informative prior distributions, some of which have been designed to deal with Heywood cases. Akaike (1987), in the process of developing a model selection criterion for factor analysis, also encountered the problem of improper solutions and proposed a standard spherical prior distribution of factor loadings and a uniform distribution for the error variances. Hirose et al. (2011) build on Akaike’s work by imposing a prior distribution only on the error variances, where the inverse of the diagonal elements of the error covariance matrix has exponential distributions. To the best of our knowledge, there has been no formal proof that such penalties prevent Heywood cases. Furthermore, naive penalization can introduce considerable finite sample bias in the estimation of the factor loadings and error variances, as illustrated in the simulation studies of Section 7.

This article introduces a maximum softly penalized likelihood (MSPL) framework for factor models. Specifically, we derive sufficient conditions on an arbitrary penalty to the log-likelihood function that guarantee that maximum penalized likelihood (MPL) estimation never results in the occurrence of Heywood cases. Furthermore, we show that the penalties proposed in Akaike (1987) and Hirose et al. (2011) satisfy those conditions, while guaranteeing key equivariance properties for factor analysis, namely, equivariance under arbitrary scaling of the data and under factor rotations. To our knowledge, this is the first proof that those two penalties can effectively deal with Heywood cases. We, then, present conditions, under which MPL estimators have the desirable asymptotic properties expected from the ML estimator, namely, consistency and asymptotic normality, under the assumption that the limit of the estimator of the variance–covariance matrix decomposes in an exploratory factor analysis fashion. In particular, we show that this is achieved by requiring that the penalization is soft enough, in a way that adapts to how information about the model parameters accumulates. We also discuss how the Akaike (1987) and Hirose et al. (2011) penalties can be adapted for soft penalization. Although our asymptotic results assume correct specification, in which case Heywood cases are sampling artifacts, our simulation studies about model selection performance in Section 7 suggest that under factor number misspecification, MSPL estimation continues to exhibit the least-false behavior expected by the ML estimator (see, e.g., White, 1982).

The remainder of this article is organized as follows. Section 2 briefly presents the factor analysis model. The proposed MPL framework is introduced in Section 3. Section 4 states our existence result of MPL estimates, which rules out the occurrence of Heywood cases, and Section 5 provides the asymptotic behavior of MPL estimators. Section 6 discusses the scaling factors for our MSPL estimators, and Section 7 provides a series of simulation studies that investigate the finite sample performance of MSPL-based estimation and inference and compares them to existing Bayesian approaches. Section 8 gives real data examples and final remarks are provided in Section 9. Proofs of all theoretical results and additional materials are provided in the Supplementary Material.

## 2 Exploratory factor analysis

The factor analysis model for a random vector of observed variables x and *q* factors (q<p) is

(2.1) $$\begin{array}{c} x=\mu +\Lambda z+\epsilon , \end{array}$$

where $\mu ={\left(\mu_{1},\cdots ,\mu_{p}\right)}^{⊤}\in {ℜ}^{p}$ , Λ is a p×q real matrix of factor loadings, $z∼\backslash operatornameN(0_{q},I_{q})$ , $\epsilon ∼\backslash operatornameN(0_{p},\Psi )$ , and z is independent of ϵ . In the latter expressions, Ψ is a p×p diagonal matrix with *j*th diagonal element $\psi_{j}>0$ , and $0_{q}$ is a vector of *q* zeros, and $I_{q}$ is the q×q identity matrix. It follows that \operatornameE(x)=μ and $\backslash operatornamevar(x)=\Sigma =\Lambda \Lambda^{⊤}+\Psi$ . The exploratory factor analysis model is identifiable only up to orthogonal rotations of the factor loadings matrix Λ . Bartholomew et al. (2011, Chapter 3) discuss approaches that resolve unidentifiability.

In the presence of realizations of *n* independent random vectors $x_{1},\cdots ,x_{n}$ , the log-likelihood function about the parameters μ and Σ of the exploratory factor analysis model is

(2.2) $$\begin{array}{c} C-\frac{n}{2}\left[\log\backslash det\left(\Sigma \right)+\backslash operatornametr\left(\Sigma^{-1}S\right)+\sum_{i=1}^{n}{\left(\bar{x}-\mu \right)}^{⊤}\Sigma^{-1}(\bar{x}-\mu )\right], \end{array}$$

where C=−nplog(2π)/2 , $\bar{x}=\sum_{i=1}^{n}x_{i}/n$ and $S=\sum_{i=1}^{n}(x_{i}-\bar{x}){\left(x_{i}-\bar{x}\right)}^{⊤}/n$ is the sample covariance matrix, assumed to be full rank. Clearly, the maximizer of (2.2) with respect to μ is $\bar{x}$ , and at that point the quadratic term in (2.2) involving $\bar{x}$ and μ vanishes. Then, the profile log-likelihood about Ψ and Λ is

(2.3) $$\begin{array}{c} \ell (\theta ;S)=C-\frac{n}{2}\left[\log\backslash det\left(\Lambda \Lambda^{⊤}+\Psi \right)+\backslash operatornametr\left.l,b,r,a,ce{\left(\Lambda \Lambda^{⊤}+\Psi \right)}^{-1}S,r,b,r,a\right.ce\right] \end{array}$$

with $\theta ={\left(\theta_{1},\cdots ,\theta_{p(q+1)}\right)}^{⊤}={\left(\lambda_{11},\cdots ,\lambda_{pq},\psi_{1},\cdots ,\psi_{p}\right)}^{⊤}$ , where $\lambda_{jk}$ and $\psi_{j}$ are the (j,k) th and (j,j) th elements of Λ and Ψ , respectively, (j=1,⋯,p;k=1,⋯,q) . Heywood cases correspond to directions $\{\theta (r){\}}_{r\in N}$ such that the value of ℓ(θ(r);S) increases but $\lim_{r\to \infty }\Psi (\theta (r))$ is no longer positive definite. Ultra-Heywood cases, that is, maxima of (2.3) with some of the variances negative, can, of course, be prevented by maximizing the log-likelihood under the constraint that $\psi_{j}>0$ . Nevertheless, this does not eliminate the possibility of at least one of the ML estimates of $\psi_{11},\cdots ,\psi_{pp}$ being exactly zero.

## 3 Maximum penalized likelihood for handling Heywood cases

A straightforward way to avoid Heywood cases is to employ an MPL estimator

(3.1) $$\begin{array}{c} \tilde{\theta }\in \backslash arg\backslash underset\theta \in \Theta \max\ell^{*}(\theta ;S), \end{array}$$

where $\ell^{*}(\theta ;S)=\ell (\theta ;S)+P^{*}(\theta ;S)$ and $\Theta =\left.l,b,r,a,ce\theta \in {ℜ}^{p(q+1)}:\theta_{m}>0,m>pq,r,b,r,a\right.ce$ , with a penalty function $P^{*}(\theta )$ that discourages estimates of $\psi_{ii}$ being zero, so that the set

$$\begin{array}{c} \backslash arg\backslash underset\theta \in \Theta \max\ell^{*}(\theta ;S)=\left.l,b,r,a,ce\bar{\theta }\in \Theta :\ell^{*}(\bar{\theta };S)=\backslash underset\theta \in \Theta \sup\ell^{*}(\theta ;S),r,b,r,a\right.ce, \end{array}$$

is nonempty. Toward constructing an MPL estimator that always exists, Akaike (1987) and Hirose et al. (2011) proposed the penalties

(3.2) $$\begin{array}{c} P^{*}(\theta )=-\frac{\rho n}{2}\backslash operatornametr\left(\Psi^{-1/2}\Lambda \Lambda^{⊤}\Psi^{-1/2}\right),\;\text{and}\;P^{*}(\theta )=-\frac{\rho n}{2}\backslash operatornametr\left(\Psi^{-1/2}S\Psi^{-1/2}\right), \end{array}$$

respectively, for ρ>0 . The penalties in (3.2) are attractive because the MPL estimates preserve two particular equivariance properties that the ML estimator has, namely, equivariance under rescaling of the response vectors and equivariance under rotations of the factor loadings. The former is desirable to justify the common practice in factor analysis of setting S in (2.3) to the sample correlation matrix, and the latter is desirable because it ensures that any post-fit rotation of the factors is still the ML estimate of the rotated factors.

To see the equivariance under rescaling of the response vectors, let $˙x_{i}=Lx_{i}$ , for a known, diagonal, invertible p×p matrix L . Then, $˙\Sigma =\backslash operatornamevar(˙x_{i})=˙\Lambda ˙\Lambda^{⊤}+˙\Psi$ , with ˙Λ=LΛ and $˙\Psi =L\Psi L^{⊤}$ , and the sample variance–covariance matrix based on $˙x_{1},\cdots ,˙x_{n}$ is $˙S=LSL^{⊤}$ . Denoting $˙\theta ={\left(˙\lambda_{11},\cdots ,˙\lambda_{pq},˙\psi_{1},\cdots ,˙\psi_{p}\right)}^{⊤}$ , the cyclic property of the trace operator and properties of the determinant for products of invertible matrices can be used to show that ℓ(˙θ;˙S)=ℓ(θ;S)+˙c , where ˙c does not depend on ˙θ . Hence, if $\hat{\Lambda }$ and $\hat{\Psi }$ are the maximizers of ℓ(θ;S) , the maximizers of ℓ(˙θ;˙S) are $L\hat{\Lambda }$ and $L\hat{\Psi }L^{⊤}$ , respectively. Similar calculations show that, for both penalties in (3.2), $P^{*}(˙\theta )=P^{*}(\theta )+˙d$ for a known constant ˙d that does not depend on ˙θ . Hence, if $\tilde{\Lambda }$ and $\tilde{\Psi }$ are the maximizers of $\ell^{*}(\theta ;S)$ , the maximizers of $\ell^{*}(˙\theta ;˙S)$ are $L\tilde{\Lambda }$ and $L\tilde{\Psi }L^{⊤}$ , respectively. The equivariance under rotations of the factors is a direct consequence of the invariance of both ℓ(θ;S) and the penalties in (3.2), when Λ is replaced by ΛQ , for an orthogonal q×q matrix Q .

Despite the above attractive equivariance properties, to our knowledge, there has been no formal proof that penalties (3.2) resolve Heywood cases. Furthermore, naive choice of ρ can introduce considerable finite-sample bias in the estimation of θ , as it is illustrated later in the simulations of Section 7.

## 4 Existence of maximum penalized likelihood estimates

Theorem 4.1 provides general conditions that ensure the existence of MPL estimates and uses them to examine the properties of the penalties (3.2).

Theorem 4.1 **(Existence of MPL estimates in factor analysis).**

Let $\Theta =\left.l,b,r,a,ce\theta \in {ℜ}^{p(q+1)}:\theta_{m}>0,m>pq,r,b,r,a\right.ce$ and $\partial \Theta =\{\theta \in {ℜ}^{p(q+1)}:\exists m>pq,\theta_{m}=0\}$ and $\Sigma (\theta )=\Lambda (\theta )\Lambda {\left(\theta \right)}^{⊤}+\Psi (\theta )$ . Assume that S has full rank and that the penalty function $P^{*}(\theta ):\Theta \to ℜ:$

1. is continuous on Θ ;
2. is bounded from above on Θ , that is, $\backslash underset\theta \in \Theta \sup P^{*}(\theta )<\infty$ ;
3. diverges to −∞ for any sequence $\{\theta (r){\}}_{r\in N}$ such that $\lim_{r\to \infty }\theta (r)\in \partial \Theta$ and $\lim_{r\to \infty }\lambda_{\min}(\Sigma (\theta (r)))>0$ , where $\lambda_{\min}(A)$ is the minimum eigenvalue of a matrix A .

Then, the set of MPL estimates $\backslash displaystyle\backslash arg\max_{\theta \in \Theta }\ell^{*}(\theta ;S)$ is non-empty.

The proof of Theorem 4.1 is in Section S2.2 of the Supplementary Material. The theorem is model-agnostic in that it only requires S to have full rank and that parameter estimation is conducted using the penalized log-likelihood function of (3.1); no assumptions about the true data generating process are made. Theorem 4.1 establishes that under conditions E1–E3 for the penalty to the log-likelihood, MPL estimation always results in estimates that are not Heywood cases, in the sense that $\tilde{\theta }$ has $\tilde{\psi_{j}}>0(j=1,\cdots ,p)$ .

The penalties by Akaike (1987) and Hirose et al. (2011) in (3.2) satisfy assumptions E1–E3 for ρ>0 , and, hence, MPL estimation using either of those results in no Heywood cases. To see that, note that matrix inversion, matrix multiplication, and trace are all continuous operations on Θ . As a result, the penalties in (3.2) are continuous and assumption E1 is satisfied. The penalties in (3.2) can be re-expressed as

(4.1) $$\begin{array}{c} P^{*}(\theta )=-\frac{\rho n}{2}\sum_{j=1}^{p}\frac{A_{jj}(\theta )}{\Psi_{jj}(\theta )}, \end{array}$$

where $A_{jj}(\theta )=S_{jj}$ for the Hirose et al. (2011) penalty, and $A_{jj}(\theta )=\lambda_{j}{\left(\theta \right)}^{⊤}\lambda_{j}(\theta )$ for the Akaike (1987) penalty, where $\lambda_{j}(\theta )$ is the *j*th row of Λ(θ) , and $C_{jk}$ denotes the (j,k) th element of the matrix C . Note that $A_{jj}(\theta )/\Psi_{jj}(\theta )\geq 0$ for both penalties. Hence, (4.1) is bounded above by zero for ρ>0 , and E2 is satisfied. Now, consider a sequence $\{\theta (r){\}}_{r\in N}$ such that $\lim_{r\to \infty }\theta (r)\in \partial \Theta$ and $\lim_{r\to \infty }\lambda_{\min}(\Sigma (\theta (r)))>0$ . Then, there exists at least one j∈{1,⋯,p} such that $\Psi_{jj}(\theta (r))\to 0$ . For A(θ)=S , the penalty (4.1) diverges to −∞ as $\Psi_{jj}(\theta )\to 0$ . For the Akaike (1987) version, $\lambda_{j}{\left(\theta (r)\right)}^{⊤}\lambda_{j}(\theta (r))$ can either diverge to ∞ or converge to a constant $c_{j}>0$ . Only the former can happen for the chosen sequence $\{\theta (r){\}}_{r\in N}$ , because, for the latter, $\lambda_{j}{\left(\theta (r)\right)}^{⊤}\lambda_{j}(\theta (r))$ would need to converge to zero at an appropriate rate, in which case $\lambda_{j}{\left(\theta (r)\right)}^{⊤}\lambda_{j}(\theta (r))+\Psi_{jj}(\theta )$ converges to zero, resulting in Σ(θ(r)) having at least one zero eigenvalue. Hence, E3 is satisfied for both the Akaike (1987) and Hirose et al. (2011) penalties.

Theorem A2 in the Supplementary Material provides an existence result under more general parameterizations of the factor analysis model, which is used for proving the consistency results in Section 5.2, and which might be useful if one wishes to impose further restrictions on the structure of Σ , as is being done, for example, in confirmatory factor analysis (see, e.g., Bartholomew et al., 2011, Chapter 8).

## 5 Asymptotics for maximum penalized likelihood

### 5.1 Consistency

To discuss the consistency of estimates for Λ,Ψ in factor analysis models, we must i) define the estimands $\Lambda_{0},\Psi_{0}$ , and $\Sigma_{0}=\Lambda_{0}\Lambda_{0}^{⊤}+\Psi_{0}$ and ii) ensure identifiability.

If the modeling assumption of Section 2 is met for matrices $\Lambda_{0}$ and $\Psi_{0}$ , then the latter are the parameter values that identify the data-generating process. This is the viewpoint taken in Kano (1983) in their consistency proofs, where they introduce and use the concept of strong identifiability.

Let B be any p×q matrix and V be any positive-definite p×p diagonal matrix and define $\Sigma =BB^{⊤}+V$ . A factor model is said to be strongly identifiable if and only if, for any ϵ>0 , there exists a δ>0 such that 
(5.1)

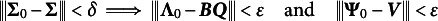

for some orthogonal matrix Q of order *q* and where 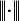 is some matrix norm. Since our results are derived in a fixed *p* and fixed *q* asymptotic regime, the particular choice of matrix and vector norm is irrelevant for the developments (see Horn & Johnson, 2012, Corollary 5.4.5 and Section S2.1 of the Supplementary Material for details). Hence, throughout, let 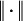 and 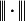 denote any vector and matrix norm, respectively.

Theorem 5.1. Assume that

1. the factor model is strongly identifiable;
2. the set of MPL estimates $\backslash arg\max_{\theta \in \Theta }\ell^{*}(\theta ;S)$ is non-empty;
3.
$P^{*}(\theta )\leq 0$ for all θ∈Θ .

Then, for any ϵ>0 , there exists a δ>0 such that 
(5.2)

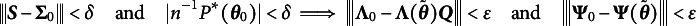

for some orthogonal q×q matrix Q .

The proof of Theorem 5.1 is in Section S2.3 of the Supplementary Material. Theorem 5.1 shows that if $S\to \Sigma_{0}$ and $n^{-1}P^{*}(\theta_{0})\to 0$ either in probability or almost surely, then the MPL estimates $\Lambda (\tilde{\theta })$ , $\Psi (\tilde{\theta })$ converge to $\Lambda_{0}$ , $\Psi_{0}$ , respectively, in probability or almost surely, up to orthogonal rotations of $\Lambda (\tilde{\theta })$ . Note that the conditions that we require of the penalty function are mild; $P^{*}(\theta )$ can be deterministic or depend on the responses, as long as it is pointwise $o_{p}(n)$ .

### $\sqrt{n}$ -consistency and asymptotic distribution

5.2

Results on the rate of consistency and the asymptotic distribution of the MPL estimator can be derived under a stronger condition on the order of the penalty than that of Theorem 5.1. In particular, we are interested in $\sqrt{n}$ -consistency of the MPL estimates, that is, 

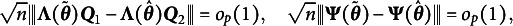

where $\tilde{\theta }$ and $\hat{\theta }$ denote the MPL and ML estimates, respectively, and $Q_{1},Q_{2}$ are appropriate sequences of orthogonal rotation matrices. Under stronger identification conditions, we can also establish results on the asymptotic distribution of $\tilde{\theta }$ and $\hat{\theta }$ .

Central to such results is the local identification restriction that the Jacobian of \operatornamevec(Σ(θ)) has full column rank at the parameter of interest $\theta_{0}$ . If the map θ↦\operatornamevec(Σ(θ)) is continuously differentiable with full column rank Jacobian at $\theta_{0}$ , then there exists an open neighborhood $U_{0}$ around $\theta_{0}$ , such that for any ε>0 , there exists a δ>0 , with 

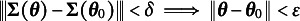

for all $\theta \in U_{0}$ . This assumption ensures that the information matrix is invertible, which is required for $\sqrt{n}$ -asymptotics. The full column rank condition N2 in Theorem 5.2 is, for example, also present in Anderson and Amemiya (1988, Theorems 2 and 3), who establish the asymptotic normal distribution of the $\sqrt{n}(\hat{\theta }-\theta_{0})$ in factor analysis models under that and additional conditions.

In the unrestricted model, where $\Theta =\{\theta \in {ℜ}^{p(q+1)}:\theta_{m}>0,m>pq\}$ , this full rank condition does not hold due to the invariance of the variance–covariance matrix under orthogonal rotations of Λ . Thus, henceforth, we focus on a restricted parameter space $\bar{\Theta }\subseteq {ℜ}^{d}$ with covariance mapping $\Sigma (\theta )=\Lambda (\theta )\Lambda {\left(\theta \right)}^{⊤}+\Psi (\theta )$ that does not allow for a rank-deficient Jacobian. We further assume that $\bar{\Theta }$ is contained in Θ in the sense that for every θ∈Θ , there exists a $\bar{\theta }\in \bar{\Theta }$ and an orthogonal rotation Q , such that $\Lambda (\theta )Q=\Lambda (\bar{\theta })$ and $\Psi (\theta )=\Psi (\bar{\theta })$ . Common parameter restrictions encountered in practice, that accommodate this requirement include requiring Λ to be upper-triangular, or requiring $\Lambda^{⊤}\Psi^{-1}\Lambda$ to be diagonal (see, e.g., Bartholomew et al., 2011, Chapter 3).

Note that the parameter vector defining the matrices $\Lambda_{0}$ and $\Psi_{0}$ need not itself be in $\bar{\Theta }$ . Rather, we assume that $\bar{\Theta }$ contains a parameter vector that corresponds to a loading matrix that can be appropriately rotated to give $\Lambda_{0}$ . Unless $\Lambda_{0}$ and $\Psi_{0}$ correspond to a parameter vector in $\bar{\Theta }$ , convergence of loading matrices is therefore naturally stated after appropriate rotation.

Theorem 5.2. Assume that

1. there exists a $\theta_{0}\in \bar{\Theta }$ such that $S\backslash oversetp⟶\Sigma (\theta_{0})$ as n→∞ ;
2.
$\Sigma (\theta_{0})$ is strongly identifiable in $\bar{\Theta }$ and the Jacobian of vec(Σ(θ)) with respect to θ has full column rank at $\theta_{0}$ ;
3. the set of MPL estimates $\backslash arg\backslash underset\theta \in \bar{\Theta }\max\{\ell^{*}(\theta ;S)\}$ is not empty;
4.
$P^{*}(\theta )=c_{n}P(\theta )$ , where P(θ) is nonpositive, deterministic, invariant under orthogonal rotations of Λ , and continuously differentiable on $\bar{\Theta }$ , with $c_{n}=o_{p}(\sqrt{n})$ positive.

Then, there exist sequences of orthogonal rotation matrices $Q_{1},Q_{2}$ such that: 
(5.3)

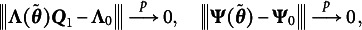

and 
(5.4)

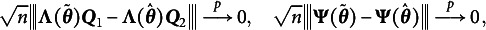

where $\hat{\theta }$ and $\tilde{\theta }$ denote two sequences of the ML and MPL estimates, respectively.

The proof of Theorem 5.2 is in Section S2.4 of the Supplementary Material. Theorem 5.2 as presented above gives conditions about the consistency of ML and MPL estimators, as well as the rate of convergence to one another. If one is interested in establishing the asymptotic distribution of the MPL estimator $\tilde{\theta }$ based on the limiting distribution of the ML estimator $\hat{\theta }$ , one can replace the strong identifiability condition on $\Lambda_{0},\Psi_{0}$ with a more stringent point-identification condition on $\theta_{0}$ :

1. for any ϵ>0 , there exists a δ>0 such that for all $\theta \in \bar{\Theta }$ , 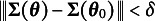 implies that 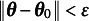 and the Jacobian of vec(Σ(θ)) with respect to θ has full column rank at $\theta_{0}$ .

In this instance, the conclusion of Theorem 5.2 is that $\tilde{\theta }\backslash oversetp⟶\theta_{0}$ and 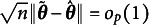. This stronger identification condition may be required when one wishes to establish the asymptotic distribution of $\tilde{\theta },\hat{\theta }$ . Slutsky’s lemma then implies that if $\sqrt{n}(\hat{\theta }-\theta_{0})$ has a normal distribution asymptotically, then $\sqrt{n}(\tilde{\theta }-\theta_{0})$ with a penalty scaled as in N4 has the same asymptotic distribution.

Note that the conditions of Theorems 5.1 and 5.2 only require that the limit of the estimator of the variance–covariance matrix S decomposes in an exploratory factor analysis fashion, and normality of errors is not essential.

## 6 Maximum softly penalized likelihood

Theorem 4.1 establishes conditions on $P^{*}(\theta )$ that ensure the existence of the MPL estimates. On the other hand, Theorems 5.1 and 5.2 involve sufficient conditions on the order of the penalty $P^{*}(\theta )$ for the consistency of the MPL estimator. Specifically, if $P^{*}(\theta )=o_{p}(\sqrt{n})$ , the respective order conditions in Theorems 5.1 and 5.2 are satisfied.

Suppose that $P^{*}(\theta )=c_{n}P(\theta )$ , where the functional part P(θ) satisfies the conditions of Theorem 4.1 for the existence of MPL estimates, and where $c_{n}>0$ is a scaling factor. One way to derive a suitable scaling factor is to consider how information about the model parameters accumulates as *n* increases. For example, we can derive a principled heuristic for $c_{n}$ by considering the exploratory factor analysis model in (2.3) under independence (i.e., Λ is a matrix of zeros). The unknown parameters are the vector of variances $\sigma_{1}^{2},\cdots \sigma_{p}^{2}$ , and the information matrix about those parameters is a diagonal matrix with *j*th diagonal element $n/(2\sigma_{j}^{4})$ . Standard results on the asymptotic distribution of the ML estimator give that $\sqrt{n/2}(s_{j}^{2}/\sigma_{j}^{2}-1)$ converges in distribution to a standard normal random variable for all j∈{1,⋯,p} . The rate of information accumulation for each coordinate is $\sqrt{n/2}$ and, hence, we can choose $c_{n}=\sqrt{2/n}$ . This choice is in line with the requirements of Theorems 5.1 and 5.2 for any exploratory factor analysis model, while ensuring that the penalization strength is asymptotically negligible. We call MSPL estimation, MPL estimation with asymptotically negligible penalties that guarantee existence and $\sqrt{n}$ -consistency. In Section 4, we showed that the conditions for the existence of the MPL estimates are satisfied for the penalties (3.2) in Akaike (1987) and Hirose et al. (2011). In particular, both penalties have the form $P^{*}(\theta )=\rho nP(\theta )/2$ , ρ>0 , P(θ)≤0, and P(θ)=O(1) . Both penalties can be adapted for MSPL estimation by setting $\rho =2\sqrt{2/n^{3}}$ .

## 7 Simulation studies

We conduct a series of simulation experiments to compare the performance of ML estimation, MPL estimation based on the penalties (3.2) with vanilla choices of ρ , and MSPL estimation. The methods are compared in terms of their ability to handle Heywood cases, frequentist properties of the estimators, and selection of the number of factors when Heywood cases are present.

Our simulation settings have been informed by the simulation-based results reported in Cooperman and Waller (2022). Specifically, Cooperman and Waller (2022) identified, through an extensive simulation study, the following causes of Heywood cases in order of importance: item-to-factor ratio, model specification (correct, fitting one factor less than the actual number, and fitting one extra factor than the actual number), sample size, and loading matrix pattern (low against high loadings on the same factor, and factors with all low loadings against factors with all high loadings).

We fix the number of factors to q=3 . We then consider sample sizes $n\in \left.l,b,r,a,ce50,100,400,r,b,r,a\right.ce$ and the item-to-factor ratios 3:1, 5:1, and 8:1, and let the loading matrix Λ vary across experimental settings. Specifically, for the loading matrix with item-to-factor ratio 3:1, we use the matrices in settings $A_{3}$ and $B_{3}$ in Table 1, which are motivated from the settings of Cooperman and Waller (2022, Table 2). Setting $A_{3}$ decreases the factor loadings sequentially, while setting $B_{3}$ assumes two strong and one weak factor. Setting $A_{5}$ for the 5:1 ratio and setting $A_{8}$ for the 8:1 ratio are defined correspondingly to $A_{3}$ , where we choose the nonzero column blocks to be (0.80,0.65,0.50,0.35,0.20) and (0.80,0.70,0.60,0.50,0.40,0.30,0.20,0.10) , respectively. For settings $B_{5}$ for the 5:1 ratio and $B_{8}$ for the 8:1 ratio, we simply repeat the non-zero loadings in Table 1 according to the item-to-factor ratio. The specific variances Ψ are set so that the diagonal elements of $\Sigma =\Lambda \Lambda^{⊤}+\Psi$ are all one.

**Table 1:** Loading matrix settings $A_{3}$ and $B_{3}$ Table 1 long description.

Item	Setting $A_{3}$				Setting $B_{3}$		
1	0.80	0	0		0.80	0	0
2	0.65	0	0		0.80	0	0
3	0.45	0	0		0.80	0	0
4	0	0.80	0		0	0.80	0
5	0	0.65	0		0	0.80	0
6	0	0.45	0		0	0.80	0
7	0	0	0.80		0	0	0.30
8	0	0	0.65		0	0	0.30
9	0	0	0.45		0	0	0.30

We compare the ML estimator with the MSPL estimators using appropriately scaled versions of the penalties (3.2) with $\rho =2\sqrt{2/n^{3}}$ based on the discussion in Section 6. We refer to those penalties as “Akaike[ $n^{-1/2}$ ]” and “Hirose[ $n^{-1/2}$ ].” We also consider MPL with the non-decaying scaling ρ=1 , which was also used in Hirose et al. (2011). We refer to those penalties as “Akaike[*n*]” and “Hirose[*n*].”

For each combination of loading matrix, sample size, and item-to-factor ratio, we draw 1,000 independent samples according to the factor analysis model (2.1) and, for each sample, we compute the estimates of (3.1). The estimates are computed by first getting MPL estimates from 100 iterations of an EM-maximization of the penalized log-likelihood, which we then use as starting values for a Newton–Raphson optimization routine. We identify Heywood cases heuristically, when at least one of the following occurs: the estimation procedure fails, the normalized gradient $\left\{\nabla \nabla^{⊤}\ell^{*}(\theta ){\}}^{-1}\nabla \ell^{*}(\theta \right)$ has at least one element with absolute value greater than $10^{-4}$ , and at least one of the estimates of $\psi_{1},\cdots ,\psi_{p}$ is less than $10^{-4}$ .

**Figure 1:**
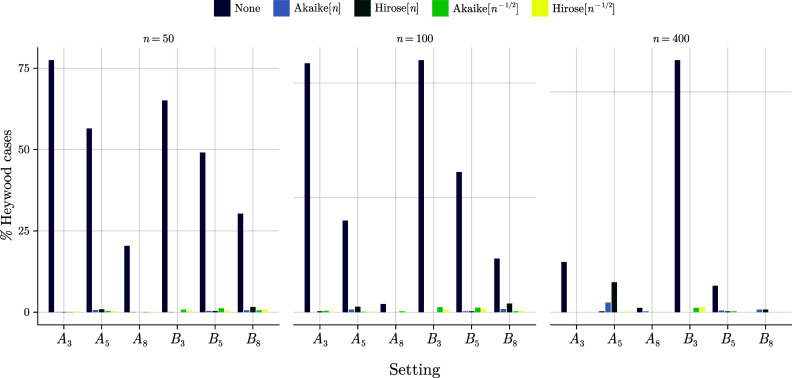
Percentage of samples (out of 1,000 ) that have been identified as Heywood cases for ML (“None”), MPL with Akaike[*n*] and Hirose[*n*] penalties, and MSPL with Akaike[ $n^{-1/2}$ ] and Hirose[ $n^{-1/2}$ ] penalties, $n\in \left.l,b,r,a,ce50,100,400,r,b,r,a\right.ce$ , and loading matrix settings $A_{3}$ , $B_{3}$ , $A_{5}$ , $B_{5}$ , $A_{8}$ , and $B_{8}$ . Figure 1 long description.

Figure 1 shows the percentage of samples that have been identified as Heywood cases. It is evident that ML estimation results in a considerable number of Heywood cases across experimental settings, while, as expected from Theorem 4.1, MPL and MSPL estimation are effective in dealing with them. The negligible fraction of estimates that are identified as Heywood cases for MPL and MSPL estimation are attributable to the heuristics we use for the identification of Heywood cases and can be eliminated by less stringent heuristics.

We evaluate the finite-sample performance of ML, MPL, and MSPL estimators in terms of bias, probability of underestimation, and root mean-squared error (RMSE), estimated excluding the samples that have been identified as Heywood cases.

**Figure 2:**
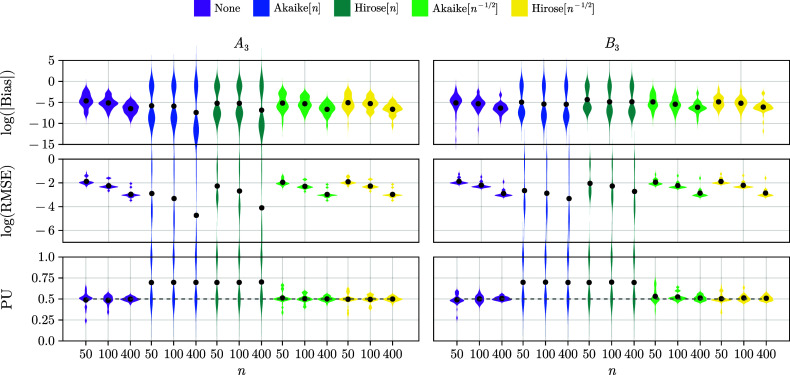
Violin plots of estimates of log(|Bias|) (top panel), log(RMSE) (middle panel), and probability of underestimation (bottom panel) for the elements of $\Lambda \Lambda^{⊤}$ , for each estimator, $n\in \left.l,b,r,a,ce50,100,400,r,b,r,a\right.ce$ , and loading matrix settings $A_{3}$ and $B_{3}$ . The average over all elements for each setting is noted with a dot. Figure 2 long description.

Figure 2 shows violin plots of coordinatewise estimates of the logarithm of the absolute bias, the logarithm of the RMSE, and the probability of underestimation of the unique elements of $\Lambda \Lambda^{⊤}$ , for each estimator, $n\in \left.l,b,r,a,ce50,100,400,r,b,r,a\right.ce$ , and loading matrix settings $A_{3}$ and $B_{3}$ . A black dot indicates the average over all coordinates in each specification. The MSPL estimates, with soft scaling of order $n^{-1/2}$ , exhibit the smallest or close to the smallest bias across all methods and a rate of decay that is in line with Theorem 5.2. Notably, the Akaike[*n*] and Hirose[*n*] penalized estimators exhibit large finite sample bias. We also see that the MSPL estimators are well calibrated, with a probability of underestimation close to 1/2 across all settings. In contrast, the Akaike[*n*] and Hirose[*n*] estimators consistently underestimate the elements of $\Lambda \Lambda^{⊤}$ . This underestimation is expected from the excessive penalization that results from using a scaling factor of order *n*. Similarly to bias, the RMSE of the MSPL estimates is the lowest or close to the minimal RMSE across all methods. The coordinatewise estimates of the logarithm of absolute bias, the logarithm of RMSE, and probability of underestimation for loading matrix settings $A_{5}$ and $B_{5}$ , and $A_{8}$ and $B_{8}$ are shown in Figures S1 and S2 in the Supplementary Material, respectively. The findings are the same as above for $A_{3}$ and $B_{3}$ .

**Figure 3:**
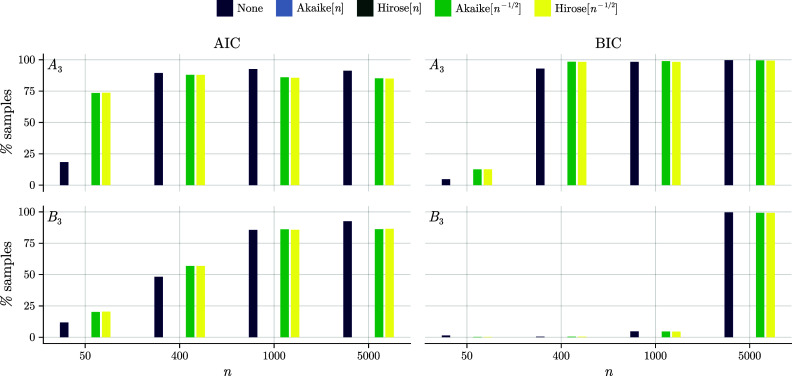
Percentage of times the model with three factors is selected for each estimator, n∈{50,400,1000,5000} , and loading matrix settings $A_{3}$ and $B_{3}$ , using AIC and BIC. The absence of vertical bars pertaining to the Akaike[*n*]- and Hirose[*n*]-based model selection procedures indicate that these methods have never selected the correct model. Figure 3 long description.

We also assess the performance of AIC and BIC selection of the number of factors based on each estimator (see Akaike, 1987 and Hirose et al., 2011 for details on these criteria for factor models) for item-to-factor ratio 3:1 with settings $A_{3}$ and $B_{3}$ for the loading matrix, and n∈{50,400,1,000,5,000} . We note that when fitting models with q∈{1,2} , the model is misspecified and the assumptions of Theorems 5.1 and 5.2 do not apply. This design choice mirrors common practitioner behavior in factor selection and motivates our inclusion of this scenario. We fit the factor analysis model (2.1) for q∈{1,⋯,5} . The AIC and BIC are computed from the unpenalized log-likelihood evaluated at the ML and MSPL estimates, respectively. We use all the samples, including those that have been identified as leading to Heywood cases, for computing AIC and BIC. Figure 3 shows the percentage of times that the model with three factors was selected with AIC and BIC at each estimator and $n\in \left.l,b,r,a,ce50,400,1,000,5,000,r,b,r,a\right.ce$ . We note that AIC- and BIC-based model selection performs as expected with the MSPL estimators with Akaike[ $n^{-1/2}$ ] and Hirose[ $n^{-1/2}$ ] penalties. BIC-based model selection selects the correct model with increasing probability as *n* increases, which is the result of the consistency of BIC-based model selection (see, e.g., Claeskens & Hjort, 2008, Chapter 4). For n=50 , model selection based on MSPL estimators is also found to outperform that based on the ML estimator, most probably due to the strong handling of Heywood cases, even in small samples. On the other hand, the MPL estimators with Akaike[*n*] and Hirose[*n*] penalties result in poor performance in both AIC- and BIC-based model selection, mainly due to the strength of the penalty.

For n<5,000 , we observe the poor performance of BIC in selecting the correct number of factors in setting $B_{3}$ across all estimators which we attribute to the two strong and one extremely weak factor (see Table 1). As Table 2 shows, BIC identifies the two strong factors in the majority of cases for small *n*, and starts identifying the weak factor more frequently slowly as *n* increases with ML or MSPL estimators.

**Table 2:** Percentage of times each number of factors has been selected using minimum BIC, for ML and MSPL with Akaike[*n*], Hirose[*n*], Akaike[ $n^{-1/2}$ ], and Hirose[ $n^{-1/2}$ ] penalties, under loading matrix setting $B_{3}$ and $n\in \left.l,b,r,a,ce50,400,1000,5000,r,b,r,a\right.ce$ Table 2 long description.

n	q	None	Akaike n	Hirose n	Akaike $n^{-1/2}$	Hirose $n^{-1/2}$
50	1	5.8	100.0	100.0	1.5	1.5
	2	92.6	0.0	0.0	98.2	98.2
	3	1.5	0.0	0.0	0.3	0.3
	4	0.1	0.0	0.0	0.0	0.0
400	1	0.0	4.7	65.1	0.0	0.0
	2	99.5	95.3	34.9	99.5	99.5
	3	0.5	0.0	0.0	0.5	0.5
	4	0.0	0.0	0.0	0.0	0.0
1,000	1	0.0	0.0	0.0	0.0	0.0
	2	95.2	100.0	100.0	95.3	95.4
	3	4.8	0.0	0.0	4.7	4.6
	4	0.0	0.0	0.0	0.0	0.0
5,000	1	0.0	0.0	0.0	0.0	0.0
	2	0.0	100.0	100.0	0.0	0.0
	3	100.0	0.0	0.0	100.0	100.0
	4	0.0	0.0	0.0	0.0	0.0

## 8 Real data examples

We estimate the factor model (2.1) using ML, and MSPL using the Akaike[ $n^{-1/2}$ ] and Hirose[ $n^{-1/2}$ ] penalties for three data sets, where Heywood cases have been encountered in published work. The data sets are i) the Davis data (Rao, 1955), which involve n=421 observations and p=9 items, ii) the Emmett data (Emmett, 1949; Lawley & Maxwell, 1971), which involve n=211 observations and p=9 items, and iii) the Maxwell data (Maxwell, 1961, and Lawley & Maxwell, 1971, p. 44), which involve n=810 observations and p=10 items. Heywood cases result in the ML estimates of the factor model (2.1) with q=2 for the Davis data, q=5 for the Emmett data, and q=4 for the Maxwell data. The three data sets have also been analyzed in Akaike (1987).

Table 3 gives the estimates of the communalities $\sum_{k=1}^{q}\lambda_{jk}^{2}(j=1,\cdots ,p)$ using ML, and MSPL with Akaike $n^{-1/2}$ and Hirose $n^{-1/2}$ penalties, across different number of factors, along with the corresponding AIC and BIC values. As expected, ML estimation can lead to Heywood cases, which manifest as atypically large estimated communalities. In contrast, and as expected, there are no Heywood cases when MSPL estimation is used, and communality estimates are reasonable with no substantial impact on AIC and BIC values. Specifically, in the Davis data set, item 1 has an atypically large communality ML estimate for q=2 , while the MSPL estimates are all within reasonable ranges. MSPL estimation also resolves the Heywood cases that result in atypically large ML communality estimates for the Emmett data for q=4 and q=5 , and the Maxwell data for q=4 . Tables S1–S3 in the Supplementary Material show the estimates of Ψ and Λ for the Emmett data, for q∈{1,⋯,5} . As is apparent, the large ML estimated communalities for q=4 and item 3, and q=5 and item 4, correspond to negative ML variance estimates for those items. In contrast, and as expected all MSPL variance estimates are positive. Notably, due to soft penalization, the ML and MSPL estimates that do not correspond to Heywood cases are similar.

**Table 3:** Estimated communalities ( $\times 10^{3}$ ) for the Davis, Emmett, and Maxwell data, using ML, and MSPL with Akaike[ $n^{-1/2}$ ] and Hirose[ $n^{-1/2}$ ] penalties, for q∈{1,⋯,5} , with AIC and BIC values Table 3 long description.

		Item											
Method	q	1	2	3	4	5	6	7	8	9	10	AIC	BIC
Davis data													
ML	1	658	661	228	168	454	800	705	434	703		1,694.69	1,767.46
	2	**14,145**	634	224	176	463	813	705	439	702		1,683.02	1,788.12
Akaike[ $n^{-1/2}$ ]	1	654	657	227	167	452	795	701	432	699		1,694.70	1,767.47
	2	877	660	226	185	467	808	698	438	696		1,684.89	1,790.00
Hirose[ $n^{-1/2}$ ]	1	658	661	228	168	454	799	705	434	703		1,694.69	1,767.46
	2	882	664	227	187	470	813	703	441	701		1,684.88	1,789.99
Emmett data													
ML	1	510	537	300	548	390	481	525	224	665		1,176.56	1,236.90
	2	538	536	332	809	592	778	597	256	782		984.56	1,071.71
	3	550	573	383	788	619	823	600	538	769		977.58	1,088.20
	4	544	556	**14,307**	797	612	800	604	737	773		986.51	1,117.23
	5	547	645	376	**8,877**	553	991	681	532	759		992.27	1,139.76
Akaike[ $n^{-1/2}$ ]	1	505	531	297	543	386	476	519	222	658		1,176.57	1,236.91
	2	532	528	329	797	585	768	590	253	770		984.59	1,071.74
	3	543	565	379	777	612	809	593	532	758		977.62	1,088.24
	4	540	557	600	789	611	797	597	615	763		986.85	1,117.58
	5	547	632	405	820	653	824	646	492	758		992.74	1,140.23
Hirose[ $n^{-1/2}$ ]	1	510	537	300	548	390	481	524	224	665		1,176.57	1,236.90
	2	538	536	333	807	592	778	597	256	780		984.57	1,071.72
	3	550	573	384	788	620	820	600	536	768		977.60	1,088.22
	4	547	565	605	800	620	807	605	619	773		986.83	1,117.56
	5	555	641	410	833	662	836	654	496	769		992.72	1,140.20
Maxwell data													
ML	1	585	230	567	306	375	159	366	143	198	114	6,442.96	6,536.88
	2	594	250	648	350	442	227	649	359	323	381	5,954.08	6,090.25
	3	631	384	694	356	586	228	664	359	325	378	5,893.17	6,066.92
	4	603	370	702	356	676	199	725	**27,024**	276	391	5,842.66	6,049.27
Akaike[ $n^{-1/2}$ ]	1	584	230	566	306	374	159	365	143	198	114	6,442.96	6,536.88
	2	593	250	647	350	442	226	648	359	322	380	5,954.08	6,090.25
	3	631	384	692	356	584	228	663	359	325	378	5,893.17	6,066.92
	4	619	382	695	363	637	225	707	903	314	405	5,847.36	6,053.98
Hirose[ $n^{-1/2}$ ]	1	584	230	567	306	374	159	366	143	198	114	6,442.96	6,536.88
	2	594	250	648	350	442	227	649	359	323	381	5,954.08	6,090.25
	3	631	384	693	356	585	228	663	359	325	378	5,893.17	6,066.92
	4	620	383	696	364	638	225	708	905	315	406	5,847.36	6,053.98

*Note*: Heywood cases are shown in bold.

## 9 Concluding remarks

In this article, we introduced a novel MSPL framework for factor analysis models to address improper solutions known as Heywood cases that frequently occur in statistical practice. Heywood cases can lead to unstable and inconclusive results related to factor loading estimates and factor scores, as well as inaccurate inferences and model selection. Our approach provides a comprehensive blueprint for constructing penalties and scaling factors that ensure the existence of estimators within the admissible parameter space and avoid the proposed ad hoc solutions in the literature. Our work focuses on exploratory factor analysis, but the proposed estimator can also be applied in a confirmatory factor analysis setting by enforcing additional constraints that practitioners might wish to impose on the model (e.g., zero loadings, equal error variances, etc.).

We provide sufficient conditions for the existence of the MPL estimator in factor analysis, together with the asymptotic properties of consistency and asymptotic normality of the MPL estimators. Additionally, we derive decay rates for the scaling of the penalty function to ensure consistency and asymptotic normality of the MSPL estimators, thus preserving the favorable asymptotic properties expected by the ML estimator. Through extensive simulation studies, we compared MSPL with appropriately scaled versions of the penalties proposed by Hirose et al. (2011), which are derived from Bayesian considerations and thus lack soft penalization by default. The MSPL estimators are found to recover the performance expected from ML theory while resolving the issues related to Heywood cases, across various model specifications, sample sizes, and item-to-factor ratios, making them a valuable tool for practical applications in exploratory and potentially confirmatory factor analysis. Our findings further reveal that naive penalties not only can undermine frequentist properties, in terms of higher bias, RMSE, and probability of underestimation, but can also have a deteriorating effect on the performance of model selection procedures. Our framework enables hypothesis testing by ensuring the existence of interior estimates in finite samples, even when the ML estimator may fail to exist, while preserving standard ML asymptotic behavior.

A limitation of our results is that they assume the probability limit of the sample covariance lies within the exploratory factor analysis model class; extending the theory to a least-false interpretation under covariance misspecification, in the spirit of White (1982), is a natural and important direction for future work. Further research directions include exploring alternative penalty functions, within the MSPL framework, for the factor analysis model (2.1) and for related models such as logistic factor analysis (see Bartholomew et al., 2011, Chapter 4). For example, in the logistic model, steep item characteristic curves lead to infinite estimates of the loadings (see, e.g., Wang & Yotebieng, 2023 for a discussion of Heywood cases in item response models for binary data). MSPL can be readily extended to handle those cases, too.

## Supporting information

10.1017/psy.2026.10092.sm001 Sterzinger et al. supplementary material Sterzinger et al. supplementary material

## Data Availability

The supplementary material is also available at https://github.com/psterzinger/FAPL, and consists of the three folders “code,” “results,” “figures,” and the Supplementary Material. The latter provides the proofs to our results and evidence from additional simulation studies and numerical examples to those presented in the main text. The “code” directory contains scripts to reproduce the numerical analyses, simulations, graphics, and tables in the main text and the Supplementary Material. The “results” and “figures” directories provide all results and figures from the numerical experiments and analyses in the main text and the Supplementary Material, respectively.

## Supplementary material

The supplementary material for this article can be found at https://doi.org/10.1017/psy.2026.10092.

## Table 1 Long description

The table has nine rows for items 1 to 9. For setting A sub 3, columns show values for each item in three positions. Items 1 to 3 have nonzero values only in the first position: 0.80, 0.65, 0.45. Items 4 to 6 have nonzero values only in the second position: 0.80, 0.65, 0.45. Items 7 to 9 have nonzero values only in the third position: 0.80, 0.65, 0.45. For setting B sub 3, items 1 to 3 have 0.80 in the first position, items 4 to 6 have 0.80 in the second position, and items 7 to 9 have 0.30 in the third position. All other values are zero. The structure highlights that for A sub 3, the nonzero values decrease from item 1 to 3 and 4 to 6, while for B sub 3, the first six items have 0.80 and the last three have 0.30 in the third position.

Navigate back to Table 1.

## Figure 1 Long description

From left to right, each panel represents a sample size: n equals 50, n equals 100, n equals 400. The y axis shows percent Heywood cases, ranging from 0 to 80. The x axis lists settings A sub 3, A sub 5, A sub 8, B sub 3, B sub 5, B sub 8. For each setting, five colored bars represent None (dark purple), Akaike open bracket n close bracket (blue), Hirose open bracket n close bracket (teal), Akaike open bracket n to the negative one half power close bracket (green), Hirose open bracket n to the negative one half power close bracket (yellow). Across all panels, the None penalty consistently yields the highest Heywood case percentages, often above 75 percent for A sub 3 and B sub 3. Penalties with Akaike and Hirose, especially with n to the negative one half power, show much lower percentages, typically below 10 percent. As n increases, the frequency of Heywood cases decreases for all penalty types, with the lowest values observed at n equals 400. The legend at the top identifies each penalty type by color.

Navigate back to Figure 1.

## Figure 2 Long description

From top-left to bottom-right, the panels are organized by loading matrix settings A sub 3 and B sub 3. Each column corresponds to a setting, with three rows for log absolute bias, log R M S E, and probability of underestimation. The x-axis in all panels is sample size n with values 50, 100, and 400. The y-axis is log absolute bias in the top row, log R M S E in the middle row, and probability of underestimation in the bottom row. Each panel contains five colored violin plots per sample size, corresponding to estimators None (purple), Akaike open bracket n close bracket (blue), Hirose open bracket n close bracket (teal), Akaike open bracket n to the negative one half power close bracket (green), and Hirose open bracket n to the negative one half power close bracket (yellow), as indicated in the legend above. Black dots mark the average over all elements for each setting. In both settings, as n increases, log absolute bias and log R M S E decrease for all estimators. Probability of underestimation centers near 0.5 for most estimators and sample sizes, with some variation. The spread of violin plots narrows with increasing n, indicating reduced variability. The dashed line at 0.5 in the bottom panels marks the reference for probability of underestimation.

Navigate back to Figure 2.

## Figure 3 Long description

From top left, the first panel shows A I C results for A sub 3. The x axis lists n values 50, 400, 1000, 5000. The y axis is percent samples from 0 to 100. For n equals 50, only the None estimator (purple) selects the correct model, at about 20 percent. For n equals 400, 1000, and 5000, Akaike open bracket n to the minus one half close bracket (green) and Hirose open bracket n to the minus one half close bracket (yellow) both reach about 75 percent, while None reaches 80 percent. Akaike open bracket n close bracket (blue) and Hirose open bracket n close bracket (teal) are absent. Top right, B I C for A sub 3: at n equals 50, all bars are near zero except None, which is about 5 percent. At n equals 400, None is about 20 percent, green and yellow are about 5 percent. For n equals 1000 and 5000, all three reach 100 percent. Bottom left, A I C for B sub 3: at n equals 50, None is about 15 percent, green and yellow about 10 percent. At n equals 400, None is about 50 percent, green and yellow about 55 percent. For n equals 1000 and 5000, all three are about 75 percent. Bottom right, B I C for B sub 3: all bars are near zero except at n equals 5000, where None, green, and yellow reach 100 percent. The legend above identifies colors for None (purple), Akaike open bracket n close bracket (blue), Hirose open bracket n close bracket (teal), Akaike open bracket n to the minus one half close bracket (green), and Hirose open bracket n to the minus one half close bracket (yellow).

Navigate back to Figure 3.

## Table 2 Long description

Starting from the top, the table is organized by sample size n in four blocks 50, 400, 1,000, and 5,000. Within each block, rows correspond to factor numbers q from 1 to 4. Columns list penalty methods None, Akaike n, Hirose n, Akaike n to the negative one half, and Hirose n to the negative one half. For n equals 50, None selects q equals 2 in 92.6 percent, Akaike n and Hirose n select q equals 1 in 100 percent, Akaike n to the negative one half and Hirose n to the negative one half select q equals 2 in 98.2 percent. For n equals 400, None selects q equals 2 in 99.5 percent, Akaike n in 95.3 percent, Hirose n in 34.9 percent, Akaike n to the negative one half and Hirose n to the negative one half in 99.5 percent. For n equals 1,000, None selects q equals 2 in 95.2 percent, Akaike n and Hirose n in 100 percent, Akaike n to the negative one half in 95.3 percent, Hirose n to the negative one half in 95.4 percent. For n equals 5,000, None selects q equals 3 in 100 percent, Akaike n and Hirose n select q equals 2 in 100 percent, Akaike n to the negative one half and Hirose n to the negative one half select q equals 3 in 100 percent. Across all n, selection is concentrated on q equals 2 or 3 depending on method and sample size, with Akaike n and Hirose n favoring lower q as n increases.

Navigate back to Table 2.

## Table 3 Long description

The table is divided into three main sections: Davis data, Emmett data, and Maxwell data. Each section contains rows for three estimation methods: ML, Akaike with n to the minus one half penalty, and Hirose with n to the minus one half penalty. For each method, results are shown for q values from 1 to 5. Columns 3 to 12 list estimated communalities for items 1 to 10, scaled by 10 to the 3 power. The final two columns report AIC and BIC values. In the Davis data, for ML with q equals 2, item 1 shows a Heywood case with a value of 14,145. In Emmett data, ML with q equals 4, item 3 is a Heywood case at 14,307, and with q equals 5, item 4 is 8,877. In Maxwell data, ML with q equals 4, item 8 is a Heywood case at 27,024. Heywood cases are indicated in bold. For each method and q, AIC and BIC values are provided, showing model fit. Communality estimates and fit indices vary across methods and datasets, with Heywood cases marking problematic estimates.

Navigate back to Table 3.
