# Supplementary material for: Maximum Softly Penalized Likelihood in Factor Analysis
Source: Psychometrika. 2026 Feb 18;91(2):494–507. doi: 10.1017/psy.2026.10092 (PMC13310817; doi:10.1017/psy.2026.10092)
Supplement: Sterzinger et al. supplementary material [file S0033312326100921sup001.pdf]

Supplementary material document for: *Maximum softly penalised likelihood in factor analysis*

Philipp Sterzinger *[1], Ioannis Kosmidis[2], and Irini Moustaki[1]

[1]London School of Economics and Political Science, Department of Statistics
[2]University of Warwick, Department of Statistics

January 26, 2026

S1 Additional numerical results

S1.1 Simulation results

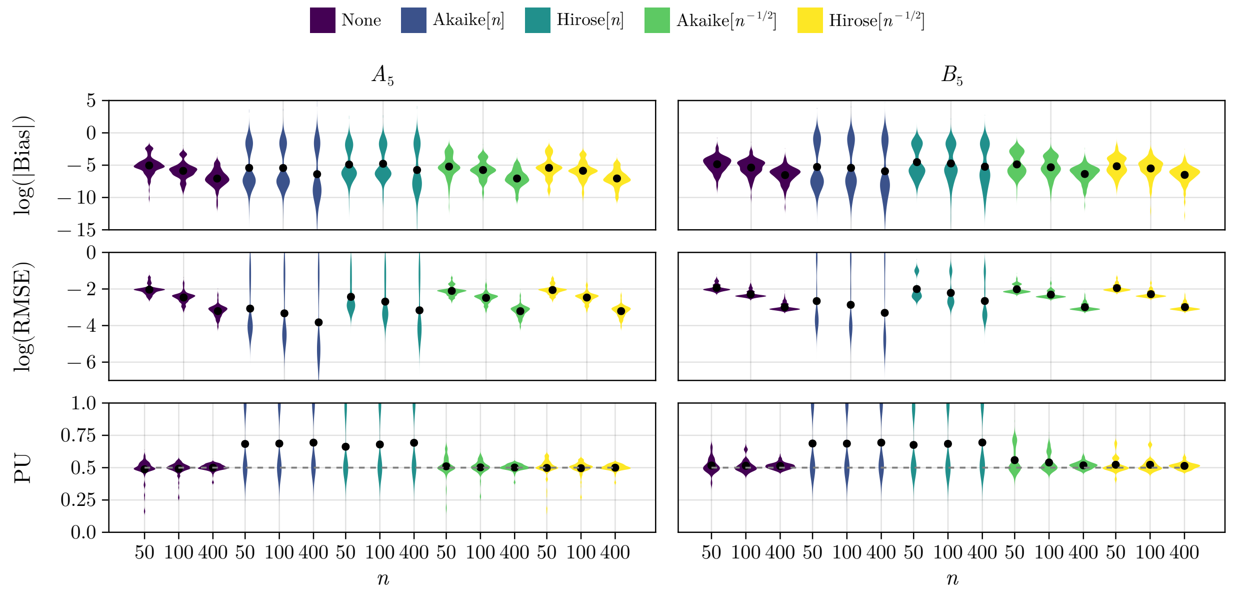

Figure S1: Violin plots of estimates of log(|Bias|) (top panel), log(RMSE) (middle panel) and probability of underestimation (bottom panel) for the elements of $\mathbf{\Lambda}\mathbf{\Lambda}^\top$, for each estimator, $n \in \{50, 100, 400\}$, and loading matrix settings A_5 and B_5. The average over all elements for each setting is noted with a dot.

*Correspondence concerning this article should be addressed to Philipp Sterzinger, Department of Statistics, London School of Economics and Political Science. E-mail: p.sterzinger@lse.ac.uk

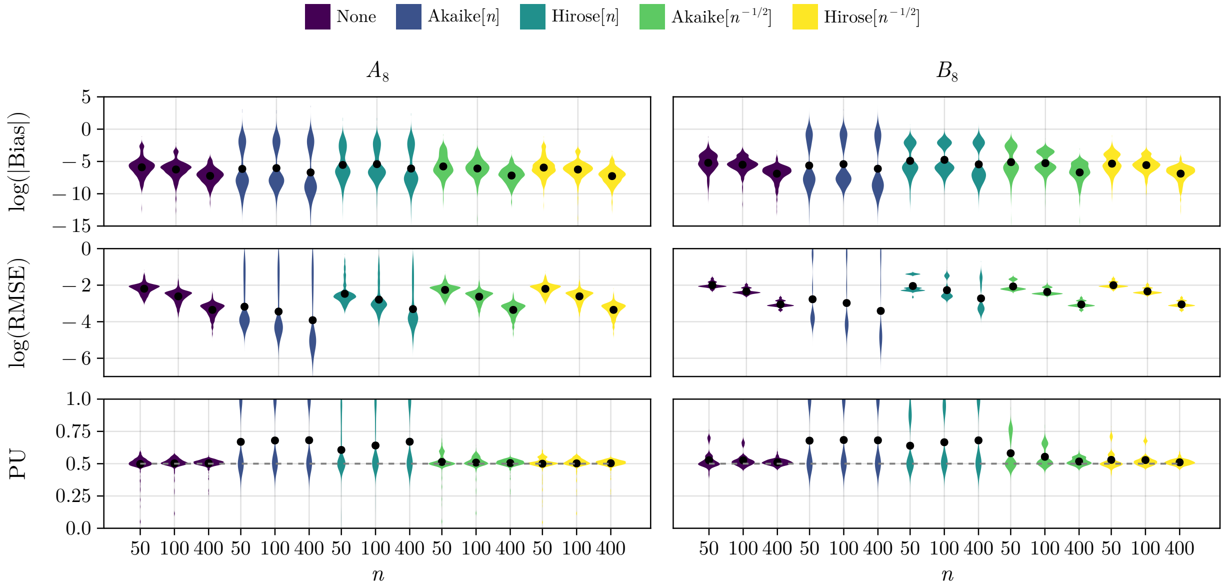

Figure S2: Violin plots of estimates of log(|Bias|) (top panel), log(RMSE) (middle panel) and probability of underestimation (bottom panel) for the elements of $\mathbf{\Lambda}\mathbf{\Lambda}^\top$, for each estimator, $n \in \{50, 100, 400\}$, and loading matrix settings A_8, and B_8. The average over all elements for each setting is noted with a dot.

S1.2 Data examples

Table S1: Estimates of $\mathbf{\Lambda}$ and $\mathbf{\Psi}$ for the Davis data using ML and MSPL with Akaike$[n^{-1/2}]$ and Hirose$[n^{-1/2}]$ penalties. A value 0.00 indicates a positive estimate that is less than 0.01, and −0.00 indicates a negative estimate that is greater than −0.01.

q	Item	ML			Akaike$[n^{-1/2}]$			Hirose$[n^{-1/2}]$		
		$\mathbf{\Lambda}_{\bullet,1}$	$\mathbf{\Lambda}_{\bullet,2}$	$\mathbf{\Psi}$	$\mathbf{\Lambda}_{\bullet,1}$	$\mathbf{\Lambda}_{\bullet,2}$	$\mathbf{\Psi}$	$\mathbf{\Lambda}_{\bullet,1}$	$\mathbf{\Lambda}_{\bullet,2}$	$\mathbf{\Psi}$
1	1	−0.81		0.34	−0.81		0.34	−0.81		0.34
	2	−0.81		0.34	−0.81		0.34	−0.81		0.34
	3	−0.48		0.77	−0.48		0.77	−0.48		0.77
	4	−0.41		0.83	−0.41		0.83	−0.41		0.83
	5	−0.67		0.55	−0.67		0.55	−0.67		0.55
	6	−0.89		0.20	−0.89		0.20	−0.89		0.20
	7	−0.84		0.29	−0.84		0.29	−0.84		0.30
	8	−0.66		0.57	−0.66		0.57	−0.66		0.57
	9	−0.84		0.30	−0.84		0.30	−0.84		0.30
2	1	−0.00	3.76	−13.14	−0.83	0.43	0.12	−0.83	0.43	0.12
	2	−0.77	0.19	0.37	−0.81	0.10	0.34	−0.81	0.10	0.34
	3	−0.46	0.11	0.78	−0.48	0.02	0.77	−0.48	0.02	0.77
	4	−0.41	0.07	0.82	−0.41	−0.14	0.81	−0.41	−0.13	0.81
	5	−0.67	0.14	0.54	−0.67	−0.11	0.53	−0.68	−0.11	0.53
	6	−0.88	0.19	0.19	−0.89	−0.10	0.19	−0.90	−0.09	0.19
	7	−0.82	0.18	0.30	−0.83	−0.05	0.30	−0.84	−0.04	0.30
	8	−0.65	0.14	0.56	−0.66	−0.09	0.56	−0.66	−0.09	0.56
	9	−0.82	0.18	0.30	−0.83	−0.05	0.30	−0.84	−0.04	0.30

Table S2: Estimates of $\mathbf{\Lambda}$ and $\mathbf{\Psi}$ for the Emmett data using ML and MSPL with Akaike[$n^{-1/2}$] and Hirose[$n^{-1/2}$] penalties. A value 0.00 indicates a positive estimate that is less than 0.01, and −0.00 indicates a negative estimate that is greater than −0.01.

q	Item	ML						Akaike[$n^{-1/2}$]						Hirose[$n^{-1/2}$]					
		$\mathbf{\Lambda}_{\bullet,1}$	$\mathbf{\Lambda}_{\bullet,2}$	$\mathbf{\Lambda}_{\bullet,3}$	$\mathbf{\Lambda}_{\bullet,4}$	$\mathbf{\Lambda}_{\bullet,5}$	$\mathbf{\Psi}$	$\mathbf{\Lambda}_{\bullet,1}$	$\mathbf{\Lambda}_{\bullet,2}$	$\mathbf{\Lambda}_{\bullet,3}$	$\mathbf{\Lambda}_{\bullet,4}$	$\mathbf{\Lambda}_{\bullet,5}$	$\mathbf{\Psi}$	$\mathbf{\Lambda}_{\bullet,1}$	$\mathbf{\Lambda}_{\bullet,2}$	$\mathbf{\Lambda}_{\bullet,3}$	$\mathbf{\Lambda}_{\bullet,4}$	$\mathbf{\Lambda}_{\bullet,5}$	$\mathbf{\Psi}$
1	1	−0.71					0.49	−0.71					0.49	−0.71					0.49
	2	−0.73					0.46	−0.73					0.46	−0.73					0.46
	3	−0.55					0.70	−0.55					0.70	−0.55					0.70
	4	−0.74					0.45	−0.74					0.45	−0.74					0.45
	5	−0.62					0.61	−0.62					0.61	−0.62					0.61
	6	−0.69					0.52	−0.69					0.52	−0.69					0.52
	7	−0.72					0.48	−0.72					0.48	−0.72					0.48
	8	−0.47					0.78	−0.47					0.78	−0.47					0.78
	9	−0.82					0.33	−0.81					0.34	−0.82					0.34
2	1	−0.73	−0.11				0.46	−0.72	−0.10				0.46	−0.73	−0.10				0.46
	2	−0.73	−0.04				0.46	−0.73	−0.03				0.46	−0.73	−0.03				0.47
	3	−0.56	−0.14				0.67	−0.56	−0.13				0.67	−0.56	−0.13				0.67
	4	−0.72	0.54				0.19	−0.71	0.54				0.19	−0.71	0.55				0.19
	5	−0.58	0.50				0.41	−0.58	0.50				0.41	−0.58	0.51				0.41
	6	−0.67	0.58				0.22	−0.66	0.58				0.22	−0.66	0.59				0.22
	7	−0.75	−0.18				0.40	−0.75	−0.18				0.40	−0.75	−0.18				0.40
	8	−0.49	−0.11				0.74	−0.49	−0.11				0.74	−0.49	−0.11				0.75
	9	−0.86	−0.19				0.22	−0.86	−0.19				0.22	−0.86	−0.18				0.22
3	1	−0.70	−0.24	0.01			0.45	−0.70	−0.23	0.01			0.45	−0.71	−0.21	0.04			0.45
	2	−0.73	−0.06	−0.20			0.43	−0.72	−0.06	−0.21			0.43	−0.73	−0.06	−0.19			0.43
	3	−0.54	−0.12	−0.27			0.62	−0.54	−0.13	−0.27			0.62	−0.55	−0.13	−0.25			0.62
	4	−0.78	0.38	0.18			0.21	−0.77	0.40	0.15			0.21	−0.77	0.43	0.13			0.21
	5	−0.65	0.43	0.06			0.38	−0.64	0.44	0.04			0.38	−0.64	0.46	0.00			0.38
	6	−0.74	0.40	0.33			0.18	−0.74	0.42	0.31			0.18	−0.73	0.46	0.28			0.18
	7	−0.72	−0.24	−0.16			0.40	−0.71	−0.24	−0.16			0.40	−0.73	−0.23	−0.13			0.40
	8	−0.47	−0.41	0.38			0.46	−0.48	−0.39	0.39			0.46	−0.49	−0.34	0.42			0.47
	9	−0.82	−0.29	−0.10			0.23	−0.82	−0.28	−0.10			0.23	−0.83	−0.26	−0.06			0.23
4	1	−0.56	−0.16	−0.10	−0.44		0.46	−0.69	−0.23	0.05	−0.10		0.45	−0.70	−0.20	0.09	−0.05		0.45
	2	−0.47	−0.00	−0.13	−0.57		0.44	−0.70	−0.11	−0.18	−0.14		0.44	−0.72	−0.12	−0.16	−0.04		0.44
	3	−0.01	−0.00	−3.78	0.02		−13.31	−0.56	−0.23	−0.45	0.18		0.40	−0.56	−0.25	−0.34	0.33		0.40
	4	−0.70	0.46	−0.09	−0.30		0.20	−0.79	0.40	0.02	0.05		0.20	−0.78	0.43	0.00	0.07		0.20
	5	−0.53	0.48	−0.07	−0.31		0.39	−0.65	0.43	−0.04	−0.08		0.38	−0.65	0.44	−0.09	−0.05		0.38
	6	−0.73	0.47	−0.07	−0.19		0.20	−0.75	0.45	0.17	0.05		0.19	−0.74	0.49	0.14	0.03		0.19
	7	−0.49	−0.18	−0.12	−0.56		0.40	−0.69	−0.27	−0.07	−0.19		0.40	−0.72	−0.27	−0.05	−0.11		0.40
	8	−0.73	−0.43	−0.05	0.12		0.26	−0.51	−0.35	0.42	0.23		0.38	−0.49	−0.26	0.53	0.17		0.38
	9	−0.60	−0.21	−0.13	−0.59		0.23	−0.80	−0.30	−0.03	−0.17		0.23	−0.83	−0.29	0.00	−0.09		0.23
5	1	−0.67	−0.26	0.08	0.15	0.05	0.45	−0.70	−0.22	0.08	0.04	−0.04	0.45	−0.70	−0.22	0.08	0.05	−0.03	0.45
	2	−0.66	−0.20	−0.16	0.16	0.34	0.35	−0.73	−0.12	−0.19	−0.07	0.22	0.36	−0.73	−0.13	−0.20	−0.06	0.22	0.36
	3	−0.51	−0.20	−0.20	0.11	0.15	0.62	−0.54	−0.21	−0.25	0.07	0.06	0.59	−0.54	−0.22	−0.25	0.07	0.06	0.59
	4	−0.03	0.00	−0.00	2.98	0.00	−7.88	−0.78	0.42	−0.01	0.19	−0.03	0.17	−0.79	0.42	−0.03	0.20	−0.02	0.17
	5	−0.57	0.33	0.08	0.23	0.25	0.45	−0.64	0.46	−0.10	−0.10	−0.11	0.34	−0.65	0.45	−0.11	−0.08	−0.11	0.34
	6	−0.63	0.35	0.46	0.26	0.44	0.00	−0.74	0.49	0.19	0.02	0.08	0.16	−0.74	0.49	0.18	0.03	0.09	0.17
	7	−0.78	−0.17	−0.10	0.14	−0.12	0.32	−0.72	−0.28	−0.08	−0.10	−0.19	0.35	−0.73	−0.28	−0.07	−0.08	−0.19	0.35
	8	−0.43	−0.39	0.42	0.09	−0.08	0.47	−0.48	−0.26	0.43	0.10	−0.04	0.50	−0.48	−0.25	0.43	0.11	−0.03	0.50
	9	−0.79	−0.31	−0.00	0.16	0.10	0.24	−0.82	−0.29	0.01	−0.09	0.02	0.23	−0.82	−0.29	0.02	−0.08	0.03	0.23

Table S3: Estimates of $\boldsymbol{\Lambda}$ and $\boldsymbol{\Psi}$ for the Maxwell data using ML and MSPL with Akaike[$n^{-1/2}$] and Hirose[$n^{-1/2}$] penalties. A value 0.00 indicates a positive estimate that is less than 0.01, and −0.00 indicates a negative estimate that is greater than −0.01.

q	Item	ML					Akaike[$n^{-1/2}$]					Hirose[$n^{-1/2}$]				
		$\boldsymbol{\Lambda}_{\bullet,1}$	$\boldsymbol{\Lambda}_{\bullet,2}$	$\boldsymbol{\Lambda}_{\bullet,3}$	$\boldsymbol{\Lambda}_{\bullet,4}$	$\boldsymbol{\Psi}$	$\boldsymbol{\Lambda}_{\bullet,1}$	$\boldsymbol{\Lambda}_{\bullet,2}$	$\boldsymbol{\Lambda}_{\bullet,3}$	$\boldsymbol{\Lambda}_{\bullet,4}$	$\boldsymbol{\Psi}$	$\boldsymbol{\Lambda}_{\bullet,1}$	$\boldsymbol{\Lambda}_{\bullet,2}$	$\boldsymbol{\Lambda}_{\bullet,3}$	$\boldsymbol{\Lambda}_{\bullet,4}$	$\boldsymbol{\Psi}$
1	1	−0.76				0.42	−0.76				0.42	−0.76				0.42
	2	−0.48				0.77	−0.48				0.77	−0.48				0.77
	3	−0.75				0.43	−0.75				0.43	−0.75				0.43
	4	−0.55				0.69	−0.55				0.69	−0.55				0.69
	5	−0.61				0.63	−0.61				0.63	−0.61				0.63
	6	−0.40				0.84	−0.40				0.84	−0.40				0.84
	7	−0.60				0.63	−0.60				0.63	−0.60				0.63
	8	−0.38				0.86	−0.38				0.86	−0.38				0.86
	9	−0.45				0.80	−0.45				0.80	−0.45				0.80
	10	−0.34				0.89	−0.34				0.89	−0.34				0.89
2	1	−0.75	0.18			0.41	−0.75	0.18			0.41	−0.77	0.06			0.41
	2	−0.47	0.18			0.75	−0.47	0.18			0.75	−0.49	0.11			0.75
	3	−0.75	0.29			0.35	−0.75	0.29			0.35	−0.79	0.17			0.35
	4	−0.54	0.25			0.65	−0.54	0.25			0.65	−0.57	0.16			0.65
	5	−0.60	0.30			0.56	−0.59	0.30			0.56	−0.63	0.20			0.56
	6	−0.41	−0.23			0.77	−0.41	−0.23			0.77	−0.37	−0.30			0.77
	7	−0.68	−0.43			0.35	−0.68	−0.43			0.35	−0.60	−0.54			0.35
	8	−0.42	−0.43			0.64	−0.42	−0.43			0.64	−0.35	−0.49			0.64
	9	−0.47	−0.31			0.68	−0.47	−0.31			0.68	−0.42	−0.38			0.68
	10	−0.39	−0.48			0.62	−0.39	−0.48			0.62	−0.31	−0.53			0.62
3	1	−0.73	0.26	0.20		0.37	−0.72	0.25	0.22		0.37	−0.72	0.24	0.22		0.37
	2	−0.47	0.23	−0.34		0.62	−0.48	0.23	−0.32		0.62	−0.48	0.23	−0.32		0.62
	3	−0.73	0.37	−0.15		0.31	−0.74	0.37	−0.12		0.31	−0.74	0.36	−0.12		0.31
	4	−0.51	0.29	−0.10		0.64	−0.52	0.29	−0.08		0.64	−0.52	0.29	−0.08		0.64
	5	−0.57	0.39	0.34		0.41	−0.56	0.37	0.36		0.42	−0.56	0.37	0.36		0.42
	6	−0.43	−0.20	−0.03		0.77	−0.43	−0.20	−0.02		0.77	−0.43	−0.20	−0.02		0.77
	7	−0.72	−0.38	0.10		0.34	−0.71	−0.38	0.11		0.34	−0.71	−0.39	0.11		0.34
	8	−0.46	−0.39	−0.04		0.64	−0.46	−0.39	−0.04		0.64	−0.46	−0.39	−0.04		0.64
	9	−0.50	−0.27	−0.04		0.67	−0.50	−0.27	−0.03		0.67	−0.50	−0.27	−0.03		0.68
	10	−0.43	−0.44	0.00		0.62	−0.43	−0.44	0.00		0.62	−0.43	−0.44	0.00		0.62
4	1	−0.74	0.14	0.19	0.03	0.40	−0.72	0.20	0.24	−0.11	0.38	−0.72	0.19	0.22	−0.12	0.38
	2	−0.46	0.32	−0.24	0.02	0.63	−0.50	0.23	−0.28	0.00	0.62	−0.50	0.22	−0.29	0.00	0.62
	3	−0.74	0.39	−0.06	0.04	0.30	−0.76	0.33	−0.07	−0.00	0.30	−0.77	0.32	−0.08	−0.01	0.30
	4	−0.51	0.30	0.00	0.03	0.64	−0.54	0.26	−0.01	0.09	0.64	−0.54	0.25	−0.02	0.09	0.64
	5	−0.61	0.21	0.51	0.03	0.32	−0.57	0.31	0.46	0.02	0.36	−0.58	0.31	0.45	0.02	0.36
	6	−0.40	−0.14	−0.11	0.07	0.80	−0.42	−0.21	−0.01	0.04	0.77	−0.42	−0.22	−0.02	0.03	0.77
	7	−0.72	−0.43	−0.12	0.09	0.28	−0.68	−0.42	0.08	−0.25	0.29	−0.67	−0.43	0.08	−0.26	0.29
	8	−0.00	−0.00	−0.00	5.20	−26.02	−0.50	−0.58	0.02	0.56	0.10	−0.50	−0.59	0.03	0.55	0.10
	9	−0.46	−0.20	−0.14	0.08	0.72	−0.48	−0.29	−0.02	0.03	0.69	−0.48	−0.29	−0.02	0.03	0.69
	10	−0.42	−0.42	−0.20	0.06	0.61	−0.39	−0.44	−0.04	−0.24	0.59	−0.38	−0.44	−0.04	−0.25	0.59

S2 Proofs

S2.1 A note on notation

The scope of this paper is a fixed p, q asymptotic regime, which means that the parameter space $\boldsymbol{\Theta} \subset \Re^d$, $d < \infty$ is a finite-dimensional real vector space. It is a fundamental property (Corollary 5.4.5 Horn and Johnson, 2012) of finite-dimensional real vector spaces that for any two norms $\|\cdot\|_\alpha$, $\|\cdot\|_\beta$, there exist positive constants C_m, C_M such that for any element $\boldsymbol{v} \in \Re^d$,

$$C_m \|\boldsymbol{v}\|_\alpha \leq \|\boldsymbol{v}\|_\beta \leq C_M \|\boldsymbol{v}\|_\alpha \,. \tag{S1}$$

This result readily generalises to matrix norms: Let $\Re^{c\times d}$, $c, d < \infty$ be a finite-dimensional matrix space. Define a map vec : $\Re^{c\times d} \to \Re^{cd}$, that is linear and bijective, e.g. by column stacking. On $\Re^{cd}$, define the vector norm

$$\|\boldsymbol{v}\| = \left|\left|\left|\mathrm{vec}^{-1}(\boldsymbol{v})\right|\right|\right| .$$

Since vec(·) was chosen to be linear and bijective, this indeed defines a vector norm and (S1) holds.

By norm equivalence, convergence to zero, divergence to infinity, and $O_p(\cdot), o_p(\cdot)$ rates of sequences of vectors or matrices do not depend on the specific choice of norm. We therefore use norms interchangeably whenever convenient. Sometimes, we wish to make use of a particular vector or matrix-norm inequality, or align our proofs with similar previous work, in which case the particular choice of norm is indicated by a subscript.

S2.2 Existence

We start by stating an existence master theorem, which we use to establish our existence results for MPL in factor analysis.

Theorem A1 (Existence master theorem). *Let $\mathcal{X} \subseteq \Re^d$, denote by $\mathrm{cl}(\mathcal{X})$ its closure and let $\partial\mathcal{X} \subseteq \mathrm{cl}(\mathcal{X})$ be a set of sequential limit points of $\mathcal{X}$ and denote $\mathrm{int}(\mathcal{X}) = \mathrm{cl}(\mathcal{X}) \setminus \partial\mathcal{X}$.*

Let $h : \mathcal{X} \to \Re$ be a function such that

A1) $h(x)$ is continuous on $\mathcal{X}$

A2) $\sup_{x\in\mathcal{X}} h(x) < \infty$

A3) For any sequence $\{x_n\}_{n\in\mathbb{N}}$, $x_n \in \mathcal{X}$ such that either $\lim_{n\to\infty} x_n \in \partial\mathcal{X}$,[1] or $\|x_n\| \to \infty$, $\lim_{n\to\infty} h(x_n) = -\infty$

Then the set of maximisers is nonempty, i.e.

$$\left\{x^* \in \mathrm{int}(\mathcal{X}) : h(x^*) = \sup_{x\in\mathcal{X}} h(x)\right\} \neq \emptyset\,.$$

Proof. Towards a contradiction, assume that supremum of h is not attained in $\mathrm{int}(\mathcal{X})$, that is, for all $x \in \mathrm{int}(\mathcal{X})$, $h(x) < H^*$, where $H^* = \sup_{x\in\mathcal{X}} h(x)$. Note that by assumption A2), $H^* \in \Re$.

Construct a sequence $\{x_n\}_{n\in\mathbb{N}}$, $x_n \in \mathcal{X}$ such that $\lim_{n\to\infty} h(x_n) = H^*$. To do this, note that for any $\varepsilon > 0$, one can find a $x \in \mathcal{X}$ for which $H^* - h(x) < \varepsilon$. If this were not the case then, there must exist a $\varepsilon > 0$ such that for all $x \in \mathcal{X}$, $H^* - \varepsilon > h(x)$, contradicting the least upper bound property of H^*. Thus, one can construct $\{x_n\}_{n\in\mathbb{N}}$ by choosing any element of the set $\{x \in \mathcal{X} : H^* - h(x) < 1/n\}$.

Next, note that $\{x_n\}_{n\in\mathbb{N}}$ must be bounded. For this, assume that on the contrary $\|x_n\| \to \infty$ as $n \to \infty$. Then by A3), $h(x_n) \to -\infty$ as $n \to \infty$. But then for every $r \in \Re$ there exists a $N \in \mathbb{N}$ such that for all $n > N$, $h(x_n) < r$. But this stands in contradiction to the construction of $\{x_n\}_{n\in\mathbb{N}}$, for which $\{h(x_n)\}_{n\in\mathbb{N}}$ ought to converge to $H^* \in \Re$.

Then, by the Bolzano-Weierstrass theorem (see for example Rudin 1976, Theorem 3.6 (b)), $\{x_n\}_{n\in\mathbb{N}}$ must contain a convergent subsequence, say $\{x_{n_k}\}_{k\in\mathbb{N}}$, with limit $x^* \in \mathrm{cl}(\mathcal{X})$ and where $\{n_k\}_{k\in\mathbb{N}} \in \mathbb{N} : n_s < n_t$ for $s < t$. Now, by construction of $\mathrm{int}(\mathcal{X}) = \mathrm{cl}(\mathcal{X}) \setminus \partial\mathcal{X}$, one of the two cases below must hold.

(i) $x^* \in \mathrm{int}(\mathcal{X})$: Then, using that the subsequential limit of a convergent sequence must equal the limit of that sequence (e.g. Rudin 1976, Definition 3.5), it follows that

$$H^* = \lim_{k\to\infty} h(x_{n_k}) = h\left(\lim_{k\to\infty} x_{n_k}\right) = h(x^*)\,,$$

where the second equality follows from continuity of $h(x)$, which holds by A1). But then there exists a $x^* \in \mathrm{int}(\mathcal{X})$ such that $h(x^*) = H^* = \sup_{x\in\mathcal{X}} h(x)$ which was assumed not to be case.

[1] Formally: $\lim_{n\to\infty} \inf_{x\in\partial\mathcal{X}} \|x_n - x\| = 0$.

(ii) $x^* \in \partial\mathcal{X}$: Then

$$H^* = h(x^*) = h\left(\lim_{k\to\infty} x_{n_k}\right) = \lim_{k\to\infty} h(x_{n_k}) = -\infty\,,$$

where the third equality follows since $h(x)$ is continuous on $\mathcal{X}$ (see for example Rudin 1976, Theorems 4.6-4.7) which holds by A1) and the last from the decay condition A3). This stands in contradiction to $H^* \in \Re$.

Since either case leads to a contradiction, the initial assumption must be false. Thus, there must exists a $x^* \in \text{int}(\mathcal{X}) : h(x^*) = H^*$.

□

Next, we state a general existence theorem for factor analysis. This result is more general than the existence Theorem 4.1 stated in the main text. In particular, it allows optimisation to be conducted in a general parameter space $\boldsymbol{\Theta} \subseteq \Re^d$, to allow for any potential constraints that one wishes to impose on the optimisation problem. Each vector $\boldsymbol{\theta} \in \boldsymbol{\Theta}$ is mapped to a symmetric, positive definite, $p \times p$ matrix through the map $\boldsymbol{\theta} \mapsto s(\boldsymbol{\theta})$.

Theorem A2. *Let $\boldsymbol{\Theta} \subseteq \Re^d$, denote by $\text{cl}(\boldsymbol{\Theta})$ its closure, $\partial\boldsymbol{\Theta} \subseteq \text{cl}(\boldsymbol{\Theta})$ a set of sequential limit points of $\boldsymbol{\Theta}$, and denote $\text{int}(\boldsymbol{\Theta}) = \text{cl}(\boldsymbol{\Theta}) \setminus \partial(\boldsymbol{\Theta})$.*

Let $s : \boldsymbol{\theta} \mapsto s(\boldsymbol{\theta})$ be a mapping from $\boldsymbol{\Theta}$ to the space of $p \times p$ positive definite and symmetric matrices and define $\ell^*(\boldsymbol{\theta}; \boldsymbol{S}) = \ell(s(\boldsymbol{\theta}); \boldsymbol{S}) + P^*(\boldsymbol{\theta})$, where $\ell(\boldsymbol{\Sigma}; \boldsymbol{S})$ is the profile log-likelihood of the EFA model in Section 2 and $\boldsymbol{S}$ is full rank.

Assume that:

A4) s and P^* are continuous on $\boldsymbol{\Theta}$

A5) For any sequence $\{\boldsymbol{\theta}_n\}_{n\in\mathbb{N}}$, $\boldsymbol{\theta}_n \in \boldsymbol{\Theta}$, such that $\|\boldsymbol{\theta}_n\| \to \infty$ as $n \to \infty$, either $P^*(\boldsymbol{\theta}) \to -\infty$ or $|||s(\boldsymbol{\theta}_n)||| \to \infty$

A6) For any sequence $\{\boldsymbol{\theta}_n\}_{n\in\mathbb{N}}$, $\boldsymbol{\theta}_n \in \boldsymbol{\Theta}$, such that (i) $\boldsymbol{\theta}_n \in \partial\boldsymbol{\Theta}$ as $n \to \infty$, and (ii) $\lambda_{\min}(s(\boldsymbol{\theta}_n)) \not\to 0$, $P^*(\boldsymbol{\theta}_n) \to -\infty$

A7) $\sup\limits_{\boldsymbol{\theta}\in\boldsymbol{\Theta}} P^*(\boldsymbol{\theta}) < \infty$

then the set of maximisers is nonempty, i.e.

$$\left\{\boldsymbol{\theta}^* \in \text{int}(\boldsymbol{\Theta}) : \ell^*(\boldsymbol{\theta}^*; \boldsymbol{S}) = \sup_{\boldsymbol{\theta}\in\boldsymbol{\Theta}} \ell^*(\boldsymbol{\theta}; \boldsymbol{S})\right\} \neq \emptyset\,.$$

Proof. We verify that the conditions of Theorem A1 are met for $\ell^*(\boldsymbol{\theta}; \boldsymbol{S})$.

A1) It is evident that $\ell(\boldsymbol{\Sigma}; \boldsymbol{S})$ is continuous on the space of $p \times p$ positive definite, symmetric matrices. By assumption A4), the map $\boldsymbol{\theta} \mapsto s(\boldsymbol{\theta})$ is continuous so that $\ell(s(\boldsymbol{\theta}); \boldsymbol{S})$ is continuous. Additionally, by A4), $P^*(\boldsymbol{\theta})$ is continuous in $\boldsymbol{\theta}$, so that $\ell^*(\boldsymbol{\theta}; \boldsymbol{S})$ is continuous in $\boldsymbol{\theta}$ on $\boldsymbol{\Theta}$.

A2) Next, let $\mathcal{S}$ be the space of $p \times p$ symmetric nonnegative definite matrices. Burg et al. (1982, Section IV) show that if $\boldsymbol{S}$ is full rank, then $\boldsymbol{S}$ is the unique maximiser of $\ell(\boldsymbol{\Sigma}; \boldsymbol{S})$ over $\mathcal{S}$. Now note that $\mathcal{S}_{\boldsymbol{\theta}} = \{\boldsymbol{\Sigma} : \boldsymbol{\Sigma} = s(\boldsymbol{\theta}), \boldsymbol{\theta} \in \boldsymbol{\Theta}\} \subseteq \mathcal{S}$. Hence,

$$\begin{aligned}
\sup_{\boldsymbol{\theta}\in\boldsymbol{\Theta}} \ell^*(\boldsymbol{\theta}; \boldsymbol{S}) &= \sup_{\boldsymbol{\theta}\in\boldsymbol{\Theta}} \{\ell(s(\boldsymbol{\theta}); \boldsymbol{S}) + P^*(\boldsymbol{\theta})\} \\
&\leq \sup_{\Sigma\in\mathcal{S}_{\boldsymbol{\theta}}} \ell(\boldsymbol{\Sigma}; \boldsymbol{S}) + \sup_{\boldsymbol{\theta}\in\boldsymbol{\Theta}} P^*(\boldsymbol{\theta}) \\
&\leq \sup_{\boldsymbol{\Sigma}\in\mathcal{S}} \ell(\boldsymbol{\Sigma}; \boldsymbol{S}) + \sup_{\boldsymbol{\theta}\in\boldsymbol{\Theta}} P^*(\boldsymbol{\theta}) \\
&= \ell(\boldsymbol{S}; \boldsymbol{S}) + \sup_{\boldsymbol{\theta}\in\boldsymbol{\Theta}} P^*(\boldsymbol{\theta}) \\
&\leq \ell(\boldsymbol{S}; \boldsymbol{S}) + C_p \\
&< \infty\,,
\end{aligned}$$

where the boundedness of $P^*(\boldsymbol{\theta})$ from above comes from assumption A7).

A3) Consider a sequence $\{\boldsymbol{\theta}_n\}_{n\in\mathbb{N}}$. (i) Assume that $\boldsymbol{\theta}_n \in \boldsymbol{\Theta}$ and $\lambda_{\min}(s(\boldsymbol{\theta}_n)) \not\to 0$, as $n \to \infty$. Then

$$\begin{aligned} \ell^*(\boldsymbol{\theta}_n; \boldsymbol{S}) &= \ell(s(\boldsymbol{\theta}_n); \boldsymbol{S}) + P^*(\boldsymbol{\theta}_n) \\ &\leq \sup_{\boldsymbol{\Sigma}\in\mathcal{S}} \{\ell(\boldsymbol{\Sigma}; \boldsymbol{S})\} + P^*(\boldsymbol{\theta}_n) \\ &= \ell(\boldsymbol{S}; \boldsymbol{S}) + P^*(\boldsymbol{\theta}_n) \\ &\to -\infty \quad \text{as}\, n \to \infty\,, \end{aligned} \tag{S2}$$

where the last line follows from A5). (ii) Assume that $\boldsymbol{\theta}_n \in \boldsymbol{\Theta}$ and $\lambda_{\min}(s(\boldsymbol{\theta}_n)) \to 0$, as $n \to \infty$. Then Burg et al. (1982, Section II) show that $\ell(s(\boldsymbol{\theta}_n); \boldsymbol{S}) \to -\infty$ as $n \to \infty$. By A7), $\ell^*(s(\boldsymbol{\theta}_n); \boldsymbol{S}) \to -\infty$ as $n \to \infty$. (iii) Assume that $\|\boldsymbol{\theta}_n\| \to \infty$ as $n \to \infty$. If $P^*(\boldsymbol{\theta}_n) \to \infty$, then the bound from (S2) establishes that $\ell^*(\boldsymbol{\theta}_n; \boldsymbol{S}) \to -\infty$. If on the other hand $|||s(\boldsymbol{\theta}_n)||| \to \infty$, Burg et al. (1982, Section II) show that $\ell(s(\boldsymbol{\theta}_n); \boldsymbol{S}) \to -\infty$ as $n \to \infty$. Therefore

$$\begin{aligned} \ell^*(\boldsymbol{\theta}_n; \boldsymbol{S}) &= \ell(s(\boldsymbol{\theta}_n); \boldsymbol{S}) + P^*(\boldsymbol{\theta}_n) \\ &\leq \ell(s(\boldsymbol{\theta}_n); \boldsymbol{S})\} + \sup_{\boldsymbol{\theta}\in\boldsymbol{\Theta}} \{P^*(\boldsymbol{\theta}_n)\} \\ &\leq \ell(s(\boldsymbol{\theta}_n); \boldsymbol{S})\} + C_p \\ &\to -\infty \quad \text{as}\, n \to \infty\,, \end{aligned}$$

□

From this result follows the existence of MPL estimates as stated in Theorem 4.1 in the main text.

Theorem A3 (Existence of MPL estimates in factor analysis)**.** *Let* $\boldsymbol{\Theta} = \left\{\boldsymbol{\theta} \in \Re^{p(q+1)} : \theta_m > 0, m > pq\right\}$ *and* $\partial\boldsymbol{\Theta} = \{\boldsymbol{\theta} \in \Re^{p(q+1)} : \exists m > pq, \theta_m = 0\}$ *and* $\boldsymbol{\Sigma}(\boldsymbol{\theta}) = \boldsymbol{\Lambda}(\boldsymbol{\theta})\boldsymbol{\Lambda}(\boldsymbol{\theta})^\top + \boldsymbol{\Psi}(\boldsymbol{\theta})$*. Assume that* $\boldsymbol{S}$ *has full rank and that the penalty function* $P^*(\boldsymbol{\theta}) : \boldsymbol{\Theta} \to \Re$

E1) is continuous on $\boldsymbol{\Theta}$*;*

E2) is bounded from above on $\boldsymbol{\Theta}$*, i.e.* $\sup_{\boldsymbol{\theta}\in\boldsymbol{\Theta}} P^*(\boldsymbol{\theta}) < \infty$*; and*

E3) diverges to $-\infty$ *for any sequence* $\{\boldsymbol{\theta}(r)\}_{r\in\mathbb{N}}$ *such that* $\lim_{r\to\infty} \boldsymbol{\theta}(r) \in \partial\boldsymbol{\Theta}$ *and* $\lim_{r\to\infty} \lambda_{\min}(\boldsymbol{\Sigma}(\boldsymbol{\theta}(r))) > 0$*, where* $\lambda_{\min}(\boldsymbol{A})$ *is the minimum eigenvalue of a matrix* $\boldsymbol{A}$*.*

Then, the set of MPL estimates $\arg\max_{\boldsymbol{\theta}\in\boldsymbol{\Theta}} \ell^*(\boldsymbol{\theta}; \boldsymbol{S})$ *is non-empty.*

Proof. We verify that the conditions of Theorem A2 are met for $\boldsymbol{\Theta} = \{\boldsymbol{\theta} \in \Re^{p(q+1)} : \forall i > pq, \theta_i > 0\}$ and $\partial\boldsymbol{\Theta} = \{\boldsymbol{\theta} \in \Re^{p(q+1)} : \exists m > pq, \theta_m = 0\}$ under E1)-E3).

First note that $\partial\boldsymbol{\Theta}$ is the boundary of $\boldsymbol{\Theta}$ and $\text{int}(\boldsymbol{\Theta}) = \boldsymbol{\Theta}$. Further recall, that $\boldsymbol{\theta} \mapsto \boldsymbol{\Sigma}(\boldsymbol{\theta})$ for $\boldsymbol{\Sigma} = \boldsymbol{\Lambda}\boldsymbol{\Lambda}^\top + \boldsymbol{\Psi}$ for $\boldsymbol{\theta} = (\theta_1, \ldots, \theta_{p(q+1)})^\top = (\lambda_{11}, \ldots, \lambda_{pq}, \psi_{11}, \ldots, \psi_{pp})^\top$, where λ_{jk} and ψ_{jj} are the (j,k)th and (j,j)th elements of $\boldsymbol{\Lambda}$ and $\boldsymbol{\Psi}$, respectively ($j = 1, \ldots, p; k = 1, \ldots, q$). Thus, for each $\boldsymbol{\theta} \in \boldsymbol{\Theta}$, $\boldsymbol{\Sigma}$ is positive definite.

It remains to verify assumptions A4)-A7).

A4) It is readily seen that $\boldsymbol{\theta} \mapsto \boldsymbol{\Sigma}(\boldsymbol{\theta})$ is continuous on $\boldsymbol{\Theta}$. Further, P^* is continuous by assumption E1).

A5) Let $\boldsymbol{\theta}(r)$ be a diverging sequence in $\boldsymbol{\Theta}$ as $r \to \infty$ and $\boldsymbol{\Sigma}(r)$ the associated sequence of variance-covariance matrices. The ith element of $\boldsymbol{\Sigma}(r)$ is given by

$$\boldsymbol{\Sigma}(r)_{ii} = \sum_{j=1} \lambda_{ij}(r)^2 + \psi_{ii}(r)\,.$$

Hence if either $|\lambda_{ij}(r)| \to \infty$ or $\psi_{ii}(r) \to \infty$ as $r \to \infty$, also $\boldsymbol{\Sigma}_{ii}(r) \to \infty$ and consequently $|||\boldsymbol{\Sigma}(r)||| \to \infty$ as required.

A6) Holds by E3).

A7) Holds by E2).

This concludes the proof. □

S2.3 Consistency

As we did for existence, and for the same reasons, we first provide a consistency result for general parameter spaces $\mathbf{\Theta}$. From this, the existence result of Theorem 5.1 follows as a corollary. These more general parameterisations are for example required for the $\sqrt{n}$-consistency results of Section 5.2 and they might be desirable if one wishes to impose further restrictions on the structure of $\mathbf{\Sigma}$.

Theorem A4. *Let $\mathbf{\Theta} \subseteq \Re^d$, denote by $cl(\mathbf{\Theta})$ its closure, $\partial\mathbf{\Theta}$ a set of sequential limit points of $\mathbf{\Theta}$ and denote by $int(\mathbf{\Theta}) = cl(\mathbf{\Theta}) \setminus \partial\mathbf{\Theta}$. Let $\mathbf{\Sigma}(\boldsymbol{\theta}) = \mathbf{\Lambda}(\boldsymbol{\theta})\mathbf{\Lambda}(\boldsymbol{\theta})^\top + \mathbf{\Psi}(\boldsymbol{\theta})$ for some maps $\boldsymbol{\theta} \mapsto \mathbf{\Lambda}(\boldsymbol{\theta})$, $\boldsymbol{\theta} \mapsto \mathbf{\Psi}(\boldsymbol{\theta})$. Finally, denote by $\ell^*(\boldsymbol{\theta}; \boldsymbol{S}) = \ell(\mathbf{\Sigma}(\boldsymbol{\theta}); \boldsymbol{S}) + P^*(\boldsymbol{\theta})$ the profile log-likelihood of the EFA model in Section 2.*

Assume that

A8) the factor model is strongly identifiable

A9) There exists a $\boldsymbol{\theta}_0 \in int(\mathbf{\Theta})$ such that $\mathbf{\Sigma}(\boldsymbol{\theta}_0) = \mathbf{\Sigma}_0$

A10)

$$\left\{\boldsymbol{\theta}^* \in \mathrm{int}(\mathbf{\Theta}) : \ell^*(\boldsymbol{\theta}^*; \boldsymbol{S}) = \sup_{\boldsymbol{\theta}\in\mathbf{\Theta}} \ell^*(\boldsymbol{\theta}; \boldsymbol{S})\right\} \neq \emptyset\,.$$

A11) $P^*(\boldsymbol{\theta}) \leq 0$ for all $\boldsymbol{\theta} \in \mathbf{\Theta}$

Then for any $\epsilon > 0$ there exists a $\delta > 0$ such that

$$|||\boldsymbol{S} - \mathbf{\Sigma}_0|||_{\max} < \delta\,, \text{and}\, |n^{-1}P^*(\boldsymbol{\theta}_0)| < \delta \implies \left|\left|\left|\mathbf{\Lambda}_0 - \mathbf{\Lambda}(\tilde{\boldsymbol{\theta}})\boldsymbol{Q}\right|\right|\right|_{\max} < \epsilon\,, \left|\left|\left|\mathbf{\Psi}_0 - \mathbf{\Psi}(\tilde{\boldsymbol{\theta}})\right|\right|\right|_{\max} < \epsilon\,,$$

for some orthogonal $q \times q$ matrix $\boldsymbol{Q}$.

Proof. The proof follows the ideas of Kano (1983) whilst accommodating the penalty function $P^*(\boldsymbol{\theta})$. First, note that the MPL estimator of (3.1) can equivalently be defined as the minimiser

$$\tilde{\boldsymbol{\theta}} = \arg\min_{\boldsymbol{\theta}\in\mathbf{\Theta}} \left\{F(\boldsymbol{S}, \mathbf{\Sigma}(\boldsymbol{\theta})) - n^{-1}P^*(\boldsymbol{\theta})\right\}\,, \tag{S3}$$

where

$$F(\mathbf{\Sigma}_1, \mathbf{\Sigma}_2) = \frac{1}{2}\left\{\mathrm{tr}\left(\mathbf{\Sigma}_2^{-1}\mathbf{\Sigma}_1\right) - p + \log\det\left(\mathbf{\Sigma}_2\right) - \log\det\left(\mathbf{\Sigma}_1\right)\right\}$$

is a criterion function that was introduced in Kano (1983).

For notational convenience, let $\tilde{\mathbf{\Sigma}} = \mathbf{\Sigma}(\tilde{\boldsymbol{\theta}}) = \mathbf{\Lambda}(\tilde{\boldsymbol{\theta}})\mathbf{\Lambda}(\tilde{\boldsymbol{\theta}})^\top + \mathbf{\Psi}(\tilde{\boldsymbol{\theta}})$. Now by (S3), and since $P^*(\boldsymbol{\theta}) \leq 0$, $|n^{-1}P^*(\boldsymbol{\theta}_0)| < \delta$, it must hold that

$$F(\boldsymbol{S}, \tilde{\mathbf{\Sigma}}) \leq F(\boldsymbol{S}, \tilde{\mathbf{\Sigma}}) - n^{-1}P^*(\tilde{\boldsymbol{\theta}}) \leq F(\boldsymbol{S}, \mathbf{\Sigma}_0) - n^{-1}P^*(\boldsymbol{\theta}_0) \leq F(\boldsymbol{S}, \mathbf{\Sigma}_0) + \delta\,. \tag{S4}$$

Now for $|||\boldsymbol{S} - \mathbf{\Sigma}_0|||_F \leq \delta$ and δ small enough, where $|||\boldsymbol{A}|||_F = \mathrm{tr}\left(\boldsymbol{A}^\top\boldsymbol{A}\right)^{1/2}$ is the Frobenius norm, it is shown in Kano (1983, equations A.2, A.4), that

$$F(\boldsymbol{S}, \mathbf{\Sigma}_0) \leq M\left|\left|\left|\mathbf{\Sigma}_0^{-1/2}\right|\right|\right|_F^4 |||\mathbf{\Sigma}_0 - \boldsymbol{S}|||_F^2 < M\left|\left|\left|\mathbf{\Sigma}_0^{-1/2}\right|\right|\right|_F^4 \delta^2\,,$$

and also

$$F(\boldsymbol{S}, \tilde{\mathbf{\Sigma}}) \geq m\left|\left|\left|\tilde{\mathbf{\Sigma}}^{-1/2}\boldsymbol{S}\tilde{\mathbf{\Sigma}}^{-1/2} - \boldsymbol{I}_p\right|\right|\right|_F^2 \geq m\left|\left|\left|\boldsymbol{S} - \tilde{\mathbf{\Sigma}}\right|\right|\right|_F^2 \left|\left|\left|\tilde{\mathbf{\Sigma}}^{1/2}\right|\right|\right|_F^{-4}\,. \tag{S5}$$

Hence, (S4)-(S5) yield

$$m\left|\left|\left|\boldsymbol{S} - \tilde{\mathbf{\Sigma}}\right|\right|\right|_F^2 \left|\left|\left|\tilde{\mathbf{\Sigma}}^{1/2}\right|\right|\right|_F^{-4} \leq m\left|\left|\left|\tilde{\mathbf{\Sigma}}^{-1/2}\boldsymbol{S}\tilde{\mathbf{\Sigma}}^{-1/2} - \boldsymbol{I}_p\right|\right|\right|_F^2 \leq \delta\left(1 + \delta M\left|\left|\left|\mathbf{\Sigma}_0^{-1/2}\right|\right|\right|_F^4\right)\,. \tag{S6}$$

It further holds by (S6) and for $|||\boldsymbol{S} - \boldsymbol{\Sigma}_0|||_{\max} \leq \delta$, δ sufficiently small, that $\left|\left|\left|\tilde{\boldsymbol{\Sigma}}^{1/2}\right|\right|\right|_F \leq C$ for some constant $C > 0$. Thus we conclude that

$$\left|\left|\left|\boldsymbol{S} - \tilde{\boldsymbol{\Sigma}}\right|\right|\right|_F^2 \leq \delta \left(1 + \delta M \left|\left|\left|\boldsymbol{\Sigma}_0^{-1/2}\right|\right|\right|_F^4\right) m^{-1} C^4 \,,$$

which can be made arbitrarily small by choosing a small enough δ. Hence,

$$\begin{aligned} \left|\left|\left|\tilde{\boldsymbol{\Sigma}} - \boldsymbol{\Sigma}_0\right|\right|\right|_{\max} &= \left|\left|\left|\tilde{\boldsymbol{\Sigma}} - \boldsymbol{S} + (\boldsymbol{S} - \boldsymbol{\Sigma}_0)\right|\right|\right|_{\max} \\ &\leq \left|\left|\left|\tilde{\boldsymbol{\Sigma}} - \boldsymbol{S}\right|\right|\right|_{\max} + |||\boldsymbol{S} - \boldsymbol{\Sigma}_0|||_{\max} \\ &\leq C'\delta + \delta \,, \end{aligned} \tag{S7}$$

where $C' > 0$ stems from (S6) and $|||\boldsymbol{A}|||_F = \sqrt{\sum_{i,j} |\boldsymbol{A}_{i,j}|^2} \geq \max_{i,j} \{|\boldsymbol{A}_{i,j}|\} = |||\boldsymbol{A}|||_{\max}$. Since the RHS in the last line of (S7) can be made arbitrarily small, the claim follows from $\tilde{\boldsymbol{\Sigma}} = \boldsymbol{\Sigma}(\tilde{\boldsymbol{\theta}}) = \boldsymbol{\Lambda}(\tilde{\boldsymbol{\theta}})\boldsymbol{\Lambda}(\tilde{\boldsymbol{\theta}})^\top + \boldsymbol{\Psi}(\tilde{\boldsymbol{\theta}})$, strong identifiability. □

Theorem A5. *Assume that*

C1) the factor model is strongly identifiable;

C2) the set of maximum penalised likelihood estimates $\arg\max_{\boldsymbol{\theta}\in\boldsymbol{\Theta}} \ell^*(\boldsymbol{\theta}; \boldsymbol{S})$ *is non-empty; and*

C3) $P^*(\boldsymbol{\theta}) \leq 0$ *for all* $\boldsymbol{\theta} \in \boldsymbol{\Theta}$.

Then, for any $\epsilon > 0$*, there exists a* $\delta > 0$ *such that*

$$|||\boldsymbol{S} - \boldsymbol{\Sigma}_0||| < \delta \quad \textit{and} \quad |n^{-1}P^*(\boldsymbol{\theta}_0)| < \delta \implies \left|\left|\left|\boldsymbol{\Lambda}_0 - \boldsymbol{\Lambda}(\tilde{\boldsymbol{\theta}})\boldsymbol{Q}\right|\right|\right| < \epsilon \quad \textit{and} \quad \left|\left|\left|\boldsymbol{\Psi}_0 - \boldsymbol{\Psi}(\tilde{\boldsymbol{\theta}})\right|\right|\right| < \epsilon \,,$$

for some orthogonal $q \times q$ *matrix* $\boldsymbol{Q}$.

Proof. Let $\boldsymbol{\Theta} = \{\boldsymbol{\theta} \in \Re^{p(q+1)} : \forall m > pq, \theta_m > 0\}$ and $\partial\boldsymbol{\Theta} = \{\boldsymbol{\theta} \in \Re^{p(q+1)} : \exists m > pq, \theta_m = 0\}$ be its boundary. The claim of the theorem immediately follows from Theorem A4. □

S2.4 $\sqrt{n}$-consistency

We first state a blanket theorem for $\sqrt{n}$-consistency of MPL estimators, from which Theorem 5.2 follows.

Theorem A6. *Let* $\tilde{\boldsymbol{\theta}}$ *and* $\hat{\boldsymbol{\theta}}$ *two sequences of maximisers of* $\ell(\boldsymbol{\theta}) + P^*(\boldsymbol{\theta})$ *and* $\ell(\boldsymbol{\theta})$ *over* $\boldsymbol{\Theta}$*, resepcetively. That is, for each n,*

$$\tilde{\boldsymbol{\theta}} \in \arg\max_{\boldsymbol{\theta}\in\boldsymbol{\Theta}} \{\ell(\boldsymbol{\theta}) + P^*(\boldsymbol{\theta})\} \,, \quad \hat{\boldsymbol{\theta}} \in \arg\max_{\boldsymbol{\theta}\in\boldsymbol{\Theta}} \{\ell(\boldsymbol{\theta})\} \,.$$

Assume that the conditions

A12) $\hat{\boldsymbol{\theta}}, \tilde{\boldsymbol{\theta}}$ *exist and converge to* $\boldsymbol{\theta}_0$*, an interior point of* $\boldsymbol{\Theta}$*, with probability approaching one as* $n \to \infty$

A13) In a closed ball $\mathcal{N}_0$ *containing* $\boldsymbol{\theta}_0$*, in the interior of* $\boldsymbol{\Theta}$*,* $\ell(\boldsymbol{\theta})$ *is twice differentiable with gradient that is continuous on an open set containing* $\mathcal{N}_0$

A14) $\sup_{\boldsymbol{\theta}\in\mathcal{N}_0} \left|\left|\left|\boldsymbol{R}_n^{-1/2}\nabla\nabla^\top\{-\ell(\boldsymbol{\theta})\}\boldsymbol{R}_n^{-1/2} - \boldsymbol{J}(\boldsymbol{\theta})\right|\right|\right| = o_p(1)$*, where* $\boldsymbol{J}(\boldsymbol{\theta})$ *is deterministic, continuous and invertible at* $\boldsymbol{\theta}_0$ *and* $\boldsymbol{R}_n$ *is a sequence of diagonal, positive definite matrices indexed by n*

A15) $P^*(\boldsymbol{\theta})$ *is differentiable on* $\mathcal{N}_0$ *around* $\boldsymbol{\theta}_0$*, and* $\sup_{\boldsymbol{\theta}\in\mathcal{N}_0} \|\boldsymbol{R}_n^{-1/2}\nabla P^*(\boldsymbol{\theta})\| = o_p(1)$

hold. Then

$$\left\|\boldsymbol{R}_n^{1/2}(\hat{\boldsymbol{\theta}} - \tilde{\boldsymbol{\theta}})\right\| = o_p(1) \,.$$

Proof. By the equivalence of norms on finite-dimensional Euclidean spaces, without loss of generality, for the remainder of this proof, let $\|\boldsymbol{v}\| = \|\boldsymbol{v}\|_\infty = \sup_{1\le i\le d} |\boldsymbol{v}_i|$ and $|||\boldsymbol{M}||| = |||\boldsymbol{M}|||_\infty$ be the corresponding operator norm.

Fix constants ϵ, δ. Define the events

$\mathcal{A}_n$: $\hat{\boldsymbol{\theta}}, \tilde{\boldsymbol{\theta}} \in \mathrm{int}(\boldsymbol{\Theta}) \cap \mathcal{N}_0 \cap B_\varepsilon(\boldsymbol{\theta}_0)$, where ε is the constant for which by continuity of $\boldsymbol{J}(\boldsymbol{\theta}_0)$, it holds that $\|\boldsymbol{\theta} - \boldsymbol{\theta}_0\| < \varepsilon$ implies $|||\boldsymbol{J}(\boldsymbol{\theta}) - \boldsymbol{J}(\boldsymbol{\theta}_0)||| < \{4|||\boldsymbol{J}(\boldsymbol{\theta}_0)^{-1}|||\}^{-1}$ and $B_\varepsilon(\boldsymbol{\theta}_0) = \{\boldsymbol{\theta} \in \boldsymbol{\Theta} : \|\boldsymbol{\theta} - \boldsymbol{\theta}_0\| < \varepsilon\}$.

$\mathcal{B}_n$: $\sup_{\boldsymbol{\theta}\in\mathcal{N}_0} |||\boldsymbol{H}_n(\boldsymbol{\theta}) - \boldsymbol{J}(\boldsymbol{\theta})||| \le \{4|||\boldsymbol{J}(\boldsymbol{\theta}_0)^{-1}|||\}^{-1}$, where $\boldsymbol{H}_n(\boldsymbol{\theta}) = \boldsymbol{R}_n^{-1/2}\nabla\nabla^\top\{-\ell(\boldsymbol{\theta})\}\boldsymbol{R}_n^{-1/2}$

$\mathcal{C}_n$: $P^*(\boldsymbol{\theta})$ is differentiable in $\mathcal{N}_0$ and $\sup_{\boldsymbol{\theta}\in\mathcal{N}_0} \|\boldsymbol{R}_n^{-1/2}\nabla P^*(\boldsymbol{\theta})\| \le \frac{\epsilon}{2|||\boldsymbol{J}(\boldsymbol{\theta}_0)^{-1}|||}$.

Assume that $\mathcal{A}_n \cap \mathcal{B}_n \cap \mathcal{C}_n$ holds. Then by $\mathcal{A}_n$, and assumptions A13) and A15),

$$\mathbf{0} = \nabla\ell(\hat{\boldsymbol{\theta}})$$
$$\mathbf{0} = \nabla\ell(\tilde{\boldsymbol{\theta}}) + \nabla P^*(\tilde{\boldsymbol{\theta}}),$$

where $\nabla\ell(\boldsymbol{\theta})$ denotes the gradient of $\ell(\boldsymbol{\theta})$ with respect to $\boldsymbol{\theta}$. Thus,

$$\nabla\ell(\hat{\boldsymbol{\theta}}) - \nabla\ell(\tilde{\boldsymbol{\theta}}) = \nabla P^*(\tilde{\boldsymbol{\theta}}).$$

By the Mean Value Theorem (see, for example, Rudin, 1976, Theorem 5.10), the ith component of the equation above can be written as

$$\left\{\nabla\nabla^\top\ell(\boldsymbol{\theta}_i^*)\right\}_{i,\bullet}^\top (\hat{\boldsymbol{\theta}} - \tilde{\boldsymbol{\theta}}) = \left\{\nabla P^*(\tilde{\boldsymbol{\theta}})\right\}_i,$$

where $\{\boldsymbol{A}\}_{i,\bullet}$ denotes the ith row of matrix $\boldsymbol{A}$ and $\boldsymbol{\theta}_i^*$ is a vector on the line segment joining $\hat{\boldsymbol{\theta}}$ and $\tilde{\boldsymbol{\theta}}$, i.e. $\boldsymbol{\theta}_i^* = c_i\hat{\boldsymbol{\theta}} + (1-c_i)\tilde{\boldsymbol{\theta}}$, $c_i \in [0,1]$. Abusing notation, let $\nabla\nabla^\top\ell(\boldsymbol{\theta}^*)$ be the matrix with rows $\left\{\nabla\nabla^\top\ell(\boldsymbol{\theta}_i^*)\right\}_{i,\bullet}$ and let $\boldsymbol{H}_n = \boldsymbol{R}_n^{-1/2}\nabla\nabla^\top\{-\ell(\boldsymbol{\theta}_i^*)\}\boldsymbol{R}_n^{-1/2}$. Then

$$\boldsymbol{J}(\boldsymbol{\theta}_0)^{-1}\boldsymbol{H}_n\boldsymbol{R}_n^{1/2}(\hat{\boldsymbol{\theta}} - \tilde{\boldsymbol{\theta}}) = -\boldsymbol{J}(\boldsymbol{\theta}_0)^{-1}\boldsymbol{R}_n^{-1/2}\nabla P^*(\tilde{\boldsymbol{\theta}}),$$

or equivalently

$$\boldsymbol{J}(\boldsymbol{\theta}_0)^{-1}\left\{\boldsymbol{H}_n - \boldsymbol{J}(\boldsymbol{\theta}_0)\right\}\boldsymbol{R}_n^{1/2}(\hat{\boldsymbol{\theta}} - \tilde{\boldsymbol{\theta}}) + \boldsymbol{R}_n^{1/2}(\hat{\boldsymbol{\theta}} - \tilde{\boldsymbol{\theta}}) = -\boldsymbol{J}(\boldsymbol{\theta}_0)^{-1}\boldsymbol{R}_n^{-1/2}\nabla P^*(\tilde{\boldsymbol{\theta}}),$$

and rearranging yields

$$\boldsymbol{R}_n^{1/2}(\hat{\boldsymbol{\theta}} - \tilde{\boldsymbol{\theta}}) = -\boldsymbol{J}(\boldsymbol{\theta}_0)^{-1}\boldsymbol{R}_n^{-1/2}\nabla P^*(\tilde{\boldsymbol{\theta}}) + \boldsymbol{J}(\boldsymbol{\theta}_0)^{-1}\left\{\boldsymbol{J}(\boldsymbol{\theta}_0) - \boldsymbol{H}_n\right\}\boldsymbol{R}_n^{1/2}(\hat{\boldsymbol{\theta}} - \tilde{\boldsymbol{\theta}}).$$

Then

$$\begin{aligned}\left\|\boldsymbol{R}_n^{1/2}(\hat{\boldsymbol{\theta}} - \tilde{\boldsymbol{\theta}})\right\| &= \left\|-\boldsymbol{J}(\boldsymbol{\theta}_0)^{-1}\boldsymbol{R}_n^{-1/2}\nabla P^*(\tilde{\boldsymbol{\theta}}) + \boldsymbol{J}(\boldsymbol{\theta}_0)^{-1}\left\{\boldsymbol{J}(\boldsymbol{\theta}_0) - \boldsymbol{H}_n\right\}\boldsymbol{R}_n^{1/2}(\hat{\boldsymbol{\theta}} - \tilde{\boldsymbol{\theta}})\right\| \\ &\le |||\boldsymbol{J}(\boldsymbol{\theta}_0)^{-1}|||\left\|\boldsymbol{R}_n^{-1/2}\nabla P^*(\tilde{\boldsymbol{\theta}})\right\| + |||\boldsymbol{J}(\boldsymbol{\theta}_0)^{-1}|||\,|||\boldsymbol{J}(\boldsymbol{\theta}_0) - \boldsymbol{H}_n|||\left\|\boldsymbol{R}_n^{1/2}(\hat{\boldsymbol{\theta}} - \tilde{\boldsymbol{\theta}})\right\|.\end{aligned} \tag{S8}$$

Now let i be the row for which the row sum of $\boldsymbol{J}(\boldsymbol{\theta}_0) - \boldsymbol{H}_n$ is maximal, then by $\mathcal{A}_n$, $\mathcal{B}_n$,

$$\begin{aligned}
|||\boldsymbol{J}(\boldsymbol{\theta}_0) - \boldsymbol{H}_n||| &= \sum_{j=1}^{d} |\boldsymbol{J}(\boldsymbol{\theta}_0)_{ij} - \boldsymbol{H}_n(\boldsymbol{\theta}_i^*)_{ij}| \\
&= \sum_{j=1}^{d} |\boldsymbol{J}(\boldsymbol{\theta}_0)_{ij} - \boldsymbol{J}(\boldsymbol{\theta}_i^*)_{ij} + \boldsymbol{J}(\boldsymbol{\theta}_i^*)_{ij} - \boldsymbol{H}_n(\boldsymbol{\theta}_i^*)_{ij}| \\
&\le \sum_{j=1}^{d} |\boldsymbol{J}(\boldsymbol{\theta}_0)_{ij} - \boldsymbol{J}(\boldsymbol{\theta}_i^*)_{ij}| + \sum_{j=1}^{d} |\boldsymbol{J}(\boldsymbol{\theta}_i^*)_{ij} - \boldsymbol{H}_n(\boldsymbol{\theta}_i^*)_{ij}| \\
&\le \sup_{1\le k\le d} \sum_{j=1}^{d} |\boldsymbol{J}(\boldsymbol{\theta}_0)_{kj} - \boldsymbol{J}(\boldsymbol{\theta}_i^*)_{kj}| + \sup_{1\le k\le d} \sum_{j=1}^{d} |\boldsymbol{J}(\boldsymbol{\theta}_i^*)_{kj} - \boldsymbol{H}_n(\boldsymbol{\theta}_i^*)_{kj}| \\
&= |||\boldsymbol{J}(\boldsymbol{\theta}_0) - \boldsymbol{J}(\boldsymbol{\theta}_i^*)||| + |||\boldsymbol{J}(\boldsymbol{\theta}_i^*) - \boldsymbol{H}_n(\boldsymbol{\theta}_i^*)||| \\
&\le |||\boldsymbol{J}(\boldsymbol{\theta}_0) - \boldsymbol{J}(\boldsymbol{\theta}_i^*)||| + \sup_{\boldsymbol{\theta}\in\mathcal{N}_0} |||\boldsymbol{J}(\boldsymbol{\theta}) - \boldsymbol{H}_n(\boldsymbol{\theta})||| \\
&\le \frac{1}{4|||\boldsymbol{J}(\boldsymbol{\theta}_0)^{-1}|||} + \frac{1}{4|||\boldsymbol{J}(\boldsymbol{\theta}_0)^{-1}|||} \\
&= \frac{1}{2|||\boldsymbol{J}(\boldsymbol{\theta}_0)^{-1}|||} \,,
\end{aligned} \tag{S9}$$

where we used that $\mathcal{A}_n$ and that $\boldsymbol{\theta}_i = c_i\hat{\boldsymbol{\theta}} + (1-c_i)\tilde{\boldsymbol{\theta}}$ so $\|\boldsymbol{\theta}_i^* - \boldsymbol{\theta}_0\| \le c_i\|\hat{\boldsymbol{\theta}} - \boldsymbol{\theta}_0\| + (1-c_i)\|\tilde{\boldsymbol{\theta}} - \boldsymbol{\theta}_0\| < \varepsilon$.

Hence, substituting (S9) into (S8), upon rearranging, yields

$$\left\|\boldsymbol{R}_n^{1/2}(\hat{\boldsymbol{\theta}} - \tilde{\boldsymbol{\theta}})\right\| \le 2|||\boldsymbol{J}(\boldsymbol{\theta}_0)^{-1}||| \left\|\boldsymbol{R}_n^{-1/2}\nabla P^*(\tilde{\boldsymbol{\theta}})\right\| ,$$

and by $\mathcal{C}_n$, we conclude that

$$\begin{aligned}
\left\|\boldsymbol{R}_n^{1/2}(\hat{\boldsymbol{\theta}} - \tilde{\boldsymbol{\theta}})\right\| &\le 2|||\boldsymbol{J}(\boldsymbol{\theta}_0)^{-1}||| \left\|\boldsymbol{R}_n^{-1/2}\nabla P^*(\tilde{\boldsymbol{\theta}})\right\| \\
&\le 2|||\boldsymbol{J}(\boldsymbol{\theta}_0)^{-1}||| \sup_{\boldsymbol{\theta}\in\mathcal{N}_0} \left\|\boldsymbol{R}_n^{-1/2}\nabla P^*(\boldsymbol{\theta})\right\| \\
&\le 2|||\boldsymbol{J}(\boldsymbol{\theta}_0)^{-1}||| \frac{\epsilon}{2|||\boldsymbol{J}(\boldsymbol{\theta}_0)^{-1}|||} \\
&= \epsilon \,.
\end{aligned}$$

Hence, we have shown that $\mathcal{A}_n \cap \mathcal{B}_n \cap \mathcal{C}_n$ implies $\|\boldsymbol{R}_n^{1/2}(\hat{\boldsymbol{\theta}} - \tilde{\boldsymbol{\theta}})\| \le \epsilon$. Therefore,

$$\Pr\left(\left\|\boldsymbol{R}_n^{1/2}(\hat{\boldsymbol{\theta}} - \tilde{\boldsymbol{\theta}})\right\| > \epsilon\right) \le 1 - \Pr(\mathcal{A}_n \cap \mathcal{B}_n \cap \mathcal{C}_n) \,.$$

By assumptions A12)-A15), there exists a N such that for all $n > N$,

$$\begin{aligned}
\Pr(\neg\mathcal{A}_n) &\le \frac{\delta}{3} \\
\Pr(\neg\mathcal{B}_n) &\le \frac{\delta}{3} \\
\Pr(\neg\mathcal{C}_n) &\le \frac{\delta}{3} \,,
\end{aligned}$$

And thus by a union bound,

$$\Pr(\mathcal{A}_n \cap \mathcal{B}_n \cap \mathcal{C}_n) = 1 - \Pr(\neg\mathcal{A}_n \cup \neg\mathcal{B}_n \cup \neg\mathcal{C}_n) \ge 1 - \delta \,,$$

so that we conclude that for any δ, ϵ, there is a N such that for all $n > N$

$$\Pr\left(\left\|\boldsymbol{R}_n^{1/2}(\hat{\boldsymbol{\theta}} - \tilde{\boldsymbol{\theta}})\right\| > \epsilon\right) \leq \delta\,.$$

□

Theorem A7. *Suppose that*

N1) there exists a $\boldsymbol{\theta}_0 \in \bar{\boldsymbol{\Theta}}$ such that $\boldsymbol{S} \xrightarrow{p} \boldsymbol{\Sigma}(\boldsymbol{\theta}_0)$ as $n \to \infty$;

N2) $\boldsymbol{\Sigma}(\boldsymbol{\theta}_0)$ is strongly identifiable in $\bar{\boldsymbol{\Theta}}$ and the Jacobian of vec$(\boldsymbol{\Sigma}(\boldsymbol{\theta}))$ with respect to $\boldsymbol{\theta}$ has full column rank at $\boldsymbol{\theta}_0$;

N3) the set of maximum penalised likelihood estimates $\arg\max_{\boldsymbol{\theta}\in\bar{\boldsymbol{\Theta}}} \{\ell^*(\boldsymbol{\theta}; \boldsymbol{S})\}$ is not empty; and

N4) $P^*(\boldsymbol{\theta}) = c_n P(\boldsymbol{\theta})$ where $P(\boldsymbol{\theta})$ is nonpositive, deterministic, invariant under orthogonal rotations of $\boldsymbol{\Lambda}$ and continuously differentiable on $\bar{\boldsymbol{\Theta}}$, with $c_n = o_p(\sqrt{n})$ positive.

Then, there exist sequences of orthogonal rotation matrices $\boldsymbol{Q}_1, \boldsymbol{Q}_2$ such that:

$$\left|\left|\left|\boldsymbol{\Lambda}(\tilde{\boldsymbol{\theta}})\boldsymbol{Q}_1 - \boldsymbol{\Lambda}_0\right|\right|\right| \xrightarrow{p} 0, \quad \left|\left|\left|\boldsymbol{\Psi}(\tilde{\boldsymbol{\theta}}) - \boldsymbol{\Psi}_0\right|\right|\right| \xrightarrow{p} 0\,,$$

and

$$\sqrt{n}\left|\left|\left|\boldsymbol{\Lambda}(\tilde{\boldsymbol{\theta}})\boldsymbol{Q}_1 - \boldsymbol{\Lambda}(\hat{\boldsymbol{\theta}})\boldsymbol{Q}_2\right|\right|\right| \xrightarrow{p} 0, \quad \sqrt{n}\left|\left|\left|\boldsymbol{\Psi}(\tilde{\boldsymbol{\theta}}) - \boldsymbol{\Psi}(\hat{\boldsymbol{\theta}})\right|\right|\right| \xrightarrow{p} 0\,,$$

where $\hat{\boldsymbol{\theta}}$ and $\tilde{\boldsymbol{\theta}}$ denote the ML and MPL estimates, respectively.

Proof. We shall prove that the conditions of Theorem A6 are met.

A12) Let $\hat{\boldsymbol{\Lambda}}, \hat{\boldsymbol{\Psi}}$ be $\boldsymbol{\Lambda}(\hat{\boldsymbol{\theta}}), \boldsymbol{\Psi}(\hat{\boldsymbol{\theta}})$, be the loading matrix and the variances of the ML unrestricted ML estimator, $\hat{\boldsymbol{\theta}}$, over $\boldsymbol{\Theta}$, respectively. Lemma 1 shows that there exists a sequence of orthogonal rotation matrices $\boldsymbol{Q}_1$, such that $\{\text{vec}(\hat{\boldsymbol{\Lambda}}\boldsymbol{Q}_1), \text{diag}(\hat{\boldsymbol{\Psi}})\} \xrightarrow{p} \boldsymbol{\theta}_0 = (\text{vec}(\boldsymbol{\Lambda}_0)^\top, \text{diag}(\boldsymbol{\Psi}_0)^\top)^\top$. Since this vector is also a maximiser of the log-likelihood restricted to $\bar{\boldsymbol{\Theta}}$, we henceforth assume that $\hat{\boldsymbol{\theta}}$ is chosen with the adequate rotation $\boldsymbol{Q}_1$ such that $\hat{\boldsymbol{\theta}} \xrightarrow{p} \boldsymbol{\theta}_0$.

Similarly, for the MPL estimator $\tilde{\boldsymbol{\theta}}$, note that assumptions N1)-N4) satisfy the conditions for consistency in Theorem A4. Hence, there exists a sequence of orthogonal rotation matrices $\boldsymbol{Q}_2$, such that $\{\text{vec}(\tilde{\boldsymbol{\Lambda}}\boldsymbol{Q}_2), \text{diag}(\tilde{\boldsymbol{\Psi}})\} \xrightarrow{p} \boldsymbol{\theta}_0$. By assumption N4), $P(\boldsymbol{\theta})$ is invariant under such orthogonal rotations, so that $\{\text{vec}(\tilde{\boldsymbol{\Lambda}}\boldsymbol{Q}_2), \text{diag}(\tilde{\boldsymbol{\Psi}})\}$ is also a maximiser of the penalised log-likelihood. Thus, henceforth assume that $\tilde{\boldsymbol{\theta}}$ chooses the adequate rotation $\boldsymbol{Q}_2$ such that $\tilde{\boldsymbol{\theta}} \xrightarrow{p} \boldsymbol{\theta}_0$.

A13) Twice-Differentiability follows by twice-differentiability of $\ell(\boldsymbol{\Sigma}; \boldsymbol{S})$ with respect to $\boldsymbol{\Sigma}$ and the construction of $\boldsymbol{\Sigma}$. Note that the partial derivatives are given by

$$\frac{\partial \ell(\boldsymbol{\Sigma}(\boldsymbol{\theta}); \boldsymbol{S})}{\partial \boldsymbol{\theta}_j} = -\frac{n}{2}\left[\text{tr}\left\{\boldsymbol{\Sigma}(\boldsymbol{\theta})^{-1}\frac{\partial \boldsymbol{\Sigma}(\boldsymbol{\theta})}{\partial \boldsymbol{\theta}_j}\right\} - \text{tr}\left\{\boldsymbol{\Sigma}(\boldsymbol{\theta})^{-1}\frac{\partial \boldsymbol{\Sigma}(\boldsymbol{\theta})}{\partial \boldsymbol{\theta}_j}\boldsymbol{\Sigma}(\boldsymbol{\theta})^{-1}\boldsymbol{S}\right\}\right]$$

which are continuous in $\boldsymbol{\Sigma}$ and $\boldsymbol{\Sigma}(\boldsymbol{\theta})$ is continuous in $\boldsymbol{\theta}$ on $\boldsymbol{\Theta}$ by N2).

A14) Note that

$$\begin{aligned}\frac{\partial^2 \ell(\boldsymbol{\Sigma}(\boldsymbol{\theta}); \boldsymbol{S})}{\partial \boldsymbol{\theta}_i \partial \boldsymbol{\theta}_j} = -\frac{n}{2}\Bigg[&-\text{tr}\left\{\boldsymbol{\Sigma}(\boldsymbol{\theta})^{-1}\frac{\partial \boldsymbol{\Sigma}(\boldsymbol{\theta})}{\partial \boldsymbol{\theta}_j}\boldsymbol{\Sigma}(\boldsymbol{\theta})^{-1}\frac{\partial \boldsymbol{\Sigma}(\boldsymbol{\theta})}{\partial \boldsymbol{\theta}_i}\right\} \\ &+ \text{tr}\left\{\boldsymbol{\Sigma}(\boldsymbol{\theta})^{-1}\frac{\partial^2 \boldsymbol{\Sigma}(\boldsymbol{\theta})}{\partial \boldsymbol{\theta}_i \partial \boldsymbol{\theta}_j}\right\} \\ &+ 2\,\text{tr}\left\{\boldsymbol{\Sigma}(\boldsymbol{\theta})^{-1}\frac{\partial \boldsymbol{\Sigma}(\boldsymbol{\theta})}{\partial \boldsymbol{\theta}_i}\boldsymbol{\Sigma}(\boldsymbol{\theta})^{-1}\frac{\partial \boldsymbol{\Sigma}(\boldsymbol{\theta})}{\partial \boldsymbol{\theta}_j}\boldsymbol{\Sigma}(\boldsymbol{\theta})^{-1}\boldsymbol{S}\right\} \\ &- \text{tr}\left\{\boldsymbol{\Sigma}(\boldsymbol{\theta})^{-1}\frac{\partial^2 \boldsymbol{\Sigma}(\boldsymbol{\theta})}{\partial \boldsymbol{\theta}_i \partial \boldsymbol{\theta}_j}\boldsymbol{\Sigma}(\boldsymbol{\theta})^{-1}\boldsymbol{S}\right\}\Bigg]\end{aligned}$$

and let the i,jth entry of $\boldsymbol{J}(\boldsymbol{\theta})$ be given by $n^{-1}\partial^2\ell(\boldsymbol{\Sigma}(\boldsymbol{\theta});\boldsymbol{\Sigma}_0)/\partial\boldsymbol{\theta}_i\partial\boldsymbol{\theta}_j$, which is clearly continuous in $\boldsymbol{\theta}$. Let $\mathcal{N}_0$ be an Euclidean ball that lies fully in the interior of $\boldsymbol{\Theta}$ and which is centred around $\boldsymbol{\theta}_0$. Finally let $\boldsymbol{R}_n = n\boldsymbol{I}_d$. Then

$$\begin{aligned}\sup_{\boldsymbol{\theta}\in\mathcal{N}_0}\left|n^{-1}\frac{\partial^2\ell(\boldsymbol{\Sigma}(\boldsymbol{\theta});\boldsymbol{S})}{\partial\boldsymbol{\theta}_i\partial\boldsymbol{\theta}_j} - \boldsymbol{J}(\boldsymbol{\theta})_{ij}\right| &= \sup_{\boldsymbol{\theta}\in\mathcal{N}_0}\left|\mathrm{tr}\left\{\boldsymbol{B}(\boldsymbol{\theta})\left[\boldsymbol{S}-\boldsymbol{\Sigma}_0\right]\right\}\right| \\ &\le \sup_{\boldsymbol{\theta}\in\mathcal{N}_0}|||\boldsymbol{B}(\boldsymbol{\theta})|||_*|||\boldsymbol{S}-\boldsymbol{\Sigma}_0|||_2 \\ &\le C|||\boldsymbol{S}-\boldsymbol{\Sigma}_0|||_2 \\ &= o_p(1)\,,\end{aligned}$$

where the second line follows by Hoelder's inequality for Schatten-norms and where $|||\boldsymbol{A}|||_*$ is the sum of all singular values of $\boldsymbol{A}$ and $|||\boldsymbol{A}|||_2$ is the spectral norm. The third line follows by the Extreme Value Theorem (see, for example, Rudin, 1976, Theorem 4.16) since for

$$\boldsymbol{B}(\boldsymbol{\theta}) = -\boldsymbol{\Sigma}(\boldsymbol{\theta})^{-1}\frac{\partial\boldsymbol{\Sigma}(\boldsymbol{\theta})}{\partial\boldsymbol{\theta}_i}\boldsymbol{\Sigma}(\boldsymbol{\theta})^{-1}\frac{\partial\boldsymbol{\Sigma}(\boldsymbol{\theta})}{\partial\boldsymbol{\theta}_j}\boldsymbol{\Sigma}(\boldsymbol{\theta})^{-1} + \frac{1}{2}\boldsymbol{\Sigma}(\boldsymbol{\theta})^{-1}\frac{\partial^2\boldsymbol{\Sigma}(\boldsymbol{\theta})}{\partial\boldsymbol{\theta}_i\partial\boldsymbol{\theta}_j}\boldsymbol{\Sigma}(\boldsymbol{\theta})^{-1}\,,$$

$|||\boldsymbol{B}(\boldsymbol{\theta})|||_*$ is continuous in $\boldsymbol{\theta}$ and $\mathcal{N}_0$ is compact. The last line follows since $\boldsymbol{S}$ converges to $\boldsymbol{\Sigma}_0$ in probability as $n\to\infty$ (N1)). Finally, to see that $\boldsymbol{J}(\boldsymbol{\theta}_0)$ is invertible, note that

$$\boldsymbol{J}(\boldsymbol{\theta}_0)_{ij} = \frac{1}{2}\,\mathrm{tr}\left\{\boldsymbol{\Sigma}_0^{-1}\frac{\partial\boldsymbol{\Sigma}(\boldsymbol{\theta}_0)}{\partial\boldsymbol{\theta}_j}\boldsymbol{\Sigma}_0^{-1}\frac{\partial\boldsymbol{\Sigma}(\boldsymbol{\theta}_0)}{\partial\boldsymbol{\theta}_i}\right\}\,.$$

Hence, we can write $\boldsymbol{J}(\boldsymbol{\theta}_0)$ as

$$\boldsymbol{J}(\boldsymbol{\theta}_0) = \frac{1}{2}\mathcal{J}^\top\left(\boldsymbol{\Sigma}_0^{-1}\otimes\boldsymbol{\Sigma}_0^{-1}\right)\mathcal{J}\,,$$

where

$$\mathcal{J} = \left[\mathrm{vec}\left(\tfrac{\partial\boldsymbol{\Sigma}(\boldsymbol{\theta}_0)}{\partial\boldsymbol{\theta}_1}\right),\quad \mathrm{vec}\left(\tfrac{\partial\boldsymbol{\Sigma}(\boldsymbol{\theta}_0)}{\partial\boldsymbol{\theta}_2}\right),\quad \cdots,\quad \mathrm{vec}\left(\tfrac{\partial\boldsymbol{\Sigma}(\boldsymbol{\theta}_0)}{\partial\boldsymbol{\theta}_d}\right)\,.\right]$$

Hence $\boldsymbol{J}(\boldsymbol{\theta}_0)$ is invertible if $\mathcal{J}$ has full column rank. This holds by our identification condition N2).

A15) Let $\mathcal{N}_0$ be as defined above. By assumption N4), $\nabla P^*(\boldsymbol{\theta})$ is continuous and hence, by the Extreme Value Theorem,

$$\begin{aligned}\sup_{\boldsymbol{\theta}\in\mathcal{N}_0}\left\|n^{-1/2}\nabla P^*(\boldsymbol{\theta})\right\| &= \frac{c_n}{\sqrt{n}}\sup_{\boldsymbol{\theta}\in\mathcal{N}_0}\|\nabla P(\boldsymbol{\theta})\| \\ &\le C\frac{c_n}{\sqrt{n}} \\ &= o_p(1)\,,\end{aligned}$$

as required.

□

Lemma 1. *In the setting of Theorem 5.2 with assumptions N1)–N2), with probability going to one, the ML estimator exists, and there exists a sequence of orthogonal rotation matrices* $\boldsymbol{Q}$ *such that*

$$\left|\left|\left|\boldsymbol{\Lambda}(\hat{\boldsymbol{\theta}})\boldsymbol{Q}-\boldsymbol{\Lambda}_0\right|\right|\right| \xrightarrow{p} 0,\quad \left|\left|\left|\boldsymbol{\Psi}(\hat{\boldsymbol{\theta}})-\boldsymbol{\Psi}_0\right|\right|\right| \xrightarrow{p} 0\,.$$

Proof. Consider the parameter space $\boldsymbol{\Theta}^* = \mathrm{vec}(\mathrm{vec}(\boldsymbol{\Theta}_1)\otimes\Re^p_{>0})$, where $\boldsymbol{\Theta}_1$ is the space of all symmetric, positive semidefinite $p\times p$ matrices with rank q. For $\boldsymbol{\theta}\in\boldsymbol{\Theta}^*$ construct the variance-covariance matrix $\boldsymbol{s}(\boldsymbol{\theta})$ by stacking the first p^2 elements of $\boldsymbol{\theta}$ into the symmetric, positive semidefinite matrix $\boldsymbol{M}$ and the remaining p entries of $\boldsymbol{\theta}$ into the diagonal matrix $\boldsymbol{U}$ and let $\boldsymbol{s}(\boldsymbol{\theta}) = \boldsymbol{M}(\boldsymbol{\theta})+\boldsymbol{U}(\boldsymbol{\theta})$.

Kano (1986, Theorems 3, 4) shows that if $\boldsymbol{\Sigma}_0$ is strongly identifiable in $\boldsymbol{\Theta}^*$ and the criterion function, which we take to be the criterion function

$$F(\boldsymbol{\Sigma},\boldsymbol{s}(\boldsymbol{\theta})) = \log\det(\boldsymbol{s}(\boldsymbol{\theta})) + \mathrm{tr}\left(\left(\boldsymbol{s}(\boldsymbol{\theta})\right)^{-1}\boldsymbol{\Sigma}\right) - p - \log\det(\boldsymbol{\Sigma})\,,$$

satisfies the condition (A2) (Kano, 1986, Section 3):

(A2) *For any* ϵ*, there is a scalar* $\delta > 0$ *such that* $|||\boldsymbol{S} - \boldsymbol{\Sigma}_0||| < \delta$ *and* $F(\boldsymbol{S}, \boldsymbol{s}(\boldsymbol{\theta})) < \delta$ *imply that* $|||\boldsymbol{s}(\boldsymbol{\theta}) - \boldsymbol{\Sigma}_0||| < \epsilon$*,*

then with probability approaching to one, the minimiser of $F(\boldsymbol{S}, \boldsymbol{s}(\boldsymbol{\theta}))$ over $\boldsymbol{\Theta}^*$, exists and is consistent for $\boldsymbol{\Lambda}_0\boldsymbol{\Lambda}_0^\top, \boldsymbol{\Psi}_0$, i.e.

$$\left|\left|\left|\boldsymbol{M}(\hat{\boldsymbol{\theta}}) - \boldsymbol{\Lambda}_0\boldsymbol{\Lambda}_0^\top\right|\right|\right| \xrightarrow{p} 0, \quad \left|\left|\left|\boldsymbol{U}(\hat{\boldsymbol{\theta}}) - \boldsymbol{\Psi}_0\right|\right|\right| \xrightarrow{p} 0\,. \tag{S10}$$

Hence, we need to show that

i) Any maximiser in $\boldsymbol{\Theta}^*$ is equivalent to a maximiser in $\boldsymbol{\Theta}$ in that $M(\hat{\boldsymbol{\theta}}) = \boldsymbol{\Lambda}(\hat{\boldsymbol{\theta}})\boldsymbol{\Lambda}(\hat{\boldsymbol{\theta}})$ for some $p \times q$ matrix $\boldsymbol{\Lambda}(\hat{\boldsymbol{\theta}})$ of rank q, that, in conjunction with $\boldsymbol{U}(\hat{\boldsymbol{\theta}})$ is a maximiser of the log-likelihood in $\boldsymbol{\Theta}$

ii) Strong identifiability in $\boldsymbol{\Theta}$ implies strong identifiability in $\boldsymbol{\Theta}^*$,

iii) condition (A2) holds, and

iv) (S10) implies that there exists a sequence of orthogonal rotation matrices $\boldsymbol{Q}$ such that $\boldsymbol{\Lambda}(\hat{\boldsymbol{\theta}})\boldsymbol{Q} \to \boldsymbol{\Lambda}_0$ in probability.

We shall prove each property in turn.
i) Note that the parameter spaces $\boldsymbol{\Theta}$ and $\boldsymbol{\Theta}^*$ have the same images under the maps $\boldsymbol{\theta} \mapsto \boldsymbol{\Sigma}(\boldsymbol{\theta}), (\boldsymbol{\theta} \in \boldsymbol{\Theta})$, $\boldsymbol{\theta} \mapsto \boldsymbol{s}(\boldsymbol{\theta}), (\boldsymbol{\theta} \in \boldsymbol{\Theta}^*)$ respectively, as any $\boldsymbol{\Lambda}\boldsymbol{\Lambda}^\top$ is a symmetric, positive semi-definite $p \times p$ matrix, and conversely any such matrix $\boldsymbol{M}$ admits a representation $\boldsymbol{M} = \boldsymbol{\Lambda}\boldsymbol{\Lambda}^\top$, where $\boldsymbol{\Lambda}$ is $p \times q$ with rank q. The property follows by noting that minimising the criterion function F is equivalent to maximising the log-likelihood function.
ii) If $\boldsymbol{\Sigma}_0 = \boldsymbol{\Lambda}_0\boldsymbol{\Lambda}_0^\top + \boldsymbol{\Psi}_0$ is strongly identifiable in $\boldsymbol{\Theta}$, then it is also strongly identifiable in $\boldsymbol{\Theta}^*$ in that for any $\epsilon > 0$, there is a $\delta > 0$ such that for $\boldsymbol{s} = \boldsymbol{M} + \boldsymbol{U}$, $\boldsymbol{M}$ symmetric positive definite $p \times p$ of rank q and $\boldsymbol{U}$ $p \times p$ diagonal positive definite,

$$|||\boldsymbol{s} - \boldsymbol{\Sigma}_0||| < \delta \implies \left|\left|\left|\boldsymbol{M} - \boldsymbol{\Lambda}_0\boldsymbol{\Lambda}_0^\top\right|\right|\right| < \epsilon, \quad |||\boldsymbol{U} - \boldsymbol{\Psi}_0||| < \epsilon\,.$$

To see this write $\boldsymbol{s} = \boldsymbol{M} + \boldsymbol{U}$ as $\boldsymbol{s} = \boldsymbol{\Lambda}\boldsymbol{\Lambda}^\top + \boldsymbol{U}$. By strong identifiability of $\boldsymbol{\Sigma}_0$ in $\boldsymbol{\Theta}$, we have that

$$|||\boldsymbol{\Lambda}\boldsymbol{Q} - \boldsymbol{\Lambda}_0||| < \epsilon, \quad |||\boldsymbol{U} - \boldsymbol{\Psi}_0||| < \epsilon\,.$$

Now let $\boldsymbol{\Delta} = \boldsymbol{\Lambda}\boldsymbol{Q} - \boldsymbol{\Lambda}_0$. Then

$$\begin{aligned}\left|\left|\left|\boldsymbol{\Lambda}\boldsymbol{\Lambda}^\top - \boldsymbol{\Lambda}_0\boldsymbol{\Lambda}_0^\top\right|\right|\right| &= \left|\left|\left|(\boldsymbol{\Lambda}_0 - \boldsymbol{\Delta})(\boldsymbol{\Lambda}_0 - \boldsymbol{\Delta})^\top - \boldsymbol{\Lambda}_0\boldsymbol{\Lambda}_0^\top\right|\right|\right| \\ &= \left|\left|\left|\boldsymbol{\Lambda}_0\boldsymbol{\Delta}^\top + \boldsymbol{\Delta}\boldsymbol{\Lambda}_0^\top + \boldsymbol{\Delta}\boldsymbol{\Delta}^\top\right|\right|\right| \\ &\leq 2\left|\left|\left|\boldsymbol{\Lambda}_0\boldsymbol{\Delta}^\top\right|\right|\right| + \left|\left|\left|\boldsymbol{\Delta}\boldsymbol{\Delta}^\top\right|\right|\right| \\ &\leq 2|||\boldsymbol{\Lambda}_0|||\epsilon + \epsilon^2\end{aligned}\,.$$

Since ϵ can be chosen arbitrarily small, this establishes strong identifiability of $\boldsymbol{\Sigma}_0$ in $\boldsymbol{\Theta}^*$.
iii) Condition (A2) is proven in Lemma 2.
iv) We can always find a $p \times q$ matrix $\boldsymbol{\Lambda}(\hat{\boldsymbol{\theta}})$ of rank q, such that $\boldsymbol{M}(\hat{\boldsymbol{\theta}}) = \boldsymbol{\Lambda}(\hat{\boldsymbol{\theta}})\boldsymbol{\Lambda}(\hat{\boldsymbol{\theta}})^\top$. What remains to be shown is that for any such sequence of $\boldsymbol{\Lambda}(\hat{\boldsymbol{\theta}})$, we can find a sequence of orthogonal rotation matrices $\boldsymbol{Q}$ such that $\left|\left|\left|\boldsymbol{\Lambda}(\hat{\boldsymbol{\theta}})\boldsymbol{Q} - \boldsymbol{\Lambda}_0\right|\right|\right| \xrightarrow{p} 0$.

From the Orthogonal Procrustes Theorem (see, for example, Golub and Van Loan 2013, Section 6.4.1) we have that for every $\boldsymbol{\Lambda}(\hat{\boldsymbol{\theta}})$, there exists an orthogonal rotation $\boldsymbol{Q}$ such that

$$\left|\left|\left|\boldsymbol{\Lambda}(\hat{\boldsymbol{\theta}})\boldsymbol{Q} - \boldsymbol{\Lambda}_0\right|\right|\right|_F^2 = \left|\left|\left|\boldsymbol{\Lambda}(\hat{\boldsymbol{\theta}})\right|\right|\right|_F^2 + |||\boldsymbol{\Lambda}_0|||_F^2 - 2\mathrm{tr}\left(\boldsymbol{R}\right)\,, \tag{S11}$$

where $\boldsymbol{R} = \mathrm{diag}(\sigma_1(\boldsymbol{\Lambda}_0^\top\boldsymbol{\Lambda}(\hat{\boldsymbol{\theta}})), \ldots, \sigma_F(\boldsymbol{\Lambda}_0^\top\boldsymbol{\Lambda}(\hat{\boldsymbol{\theta}})))$ is the diagonal matrix of singular values of $\boldsymbol{\Lambda}_0^\top\boldsymbol{\Lambda}(\hat{\boldsymbol{\theta}})$.

Now by (S11), $\left|\!\left|\!\left|\boldsymbol{\Lambda}(\hat{\boldsymbol{\theta}})\right|\!\right|\!\right|_F^2$ converges to $|||\boldsymbol{\Lambda}_0|||_F^2$ in probability. To show that the singular values $\sigma_i(\boldsymbol{\Lambda}_0^\top \boldsymbol{\Lambda}(\hat{\boldsymbol{\theta}}))$ converge to $\sigma_i(\boldsymbol{\Lambda}_0^\top \boldsymbol{\Lambda}_0)$, note that these singular values are the square roots of the eigenvalues of $\boldsymbol{T}_n = \boldsymbol{\Lambda}_0^\top \boldsymbol{\Lambda}(\hat{\boldsymbol{\theta}})\boldsymbol{\Lambda}(\hat{\boldsymbol{\theta}})^\top \boldsymbol{\Lambda}_0$. Let $\boldsymbol{T} = \boldsymbol{\Lambda}_0^\top \boldsymbol{\Lambda}_0 \boldsymbol{\Lambda}_0^\top \boldsymbol{\Lambda}_0$. Then

$$\begin{aligned} |||\boldsymbol{T}_n - \boldsymbol{T}|||_2 &= \left|\!\left|\!\left|\boldsymbol{\Lambda}_0(\boldsymbol{\Lambda}(\hat{\boldsymbol{\theta}})\boldsymbol{\Lambda}(\hat{\boldsymbol{\theta}})^\top - \boldsymbol{\Lambda}_0\boldsymbol{\Lambda}_0^\top)\boldsymbol{\Lambda}_0^\top\right|\!\right|\!\right|_2 \\ &\le |||\boldsymbol{\Lambda}_0|||_2 \left|\!\left|\!\left|\boldsymbol{\Lambda}(\hat{\boldsymbol{\theta}})\boldsymbol{\Lambda}(\hat{\boldsymbol{\theta}})^\top - \boldsymbol{\Lambda}_0\boldsymbol{\Lambda}_0^\top\right|\!\right|\!\right|_2 \\ &\xrightarrow{p} 0 \end{aligned} .$$

By Weyl's inequality (see, for example, Golub and Van Loan 2013, Corollary 8.16),

$$\left|\lambda_i(\boldsymbol{T}_n) - \lambda_i(\boldsymbol{T})\right| \le |||\boldsymbol{T}_n - \boldsymbol{T}|||_2 \xrightarrow{p} 0, \quad (i = 1, \dots, q) .$$

Thus the singular values $\sigma_i(\boldsymbol{\Lambda}_0^\top \boldsymbol{\Lambda}(\hat{\boldsymbol{\theta}}))$, which are the square roots of $\lambda_i(\boldsymbol{T}_n)$, converge to $\sigma_i(\boldsymbol{\Lambda}_0^\top \boldsymbol{\Lambda}_0)$, the square roots of $\lambda_i(\boldsymbol{T})$. Therefore

$$\operatorname{tr}(\boldsymbol{R}) \xrightarrow{p} \sum_{i=1}^{q} \sigma_i(\boldsymbol{\Lambda}_0^\top \boldsymbol{\Lambda}_0) = |||\boldsymbol{\Lambda}_0|||_F^2 ,$$

and thus by (S11) indeed

$$\left|\!\left|\!\left|\boldsymbol{\Lambda}(\hat{\boldsymbol{\theta}})\boldsymbol{Q} - \boldsymbol{\Lambda}_0\right|\!\right|\!\right|_F^2 \xrightarrow{p} 0 .$$

□

Lemma 2. *Let $\boldsymbol{\Sigma}_0$ be a $p \times p$ positive definite matrix and define, for $\boldsymbol{\Sigma}_1, \boldsymbol{\Sigma}_2$, also $p \times p$ positive definite,*

$$F(\boldsymbol{\Sigma}_1, \boldsymbol{\Sigma}_2) = \log\det(\boldsymbol{\Sigma}_2) + \operatorname{tr}(\boldsymbol{\Sigma}_2^{-1}\boldsymbol{\Sigma}_1) - p - \log\det(\boldsymbol{\Sigma}_1) .$$

Then for every $\varepsilon > 0$ there exists $\delta > 0$ such that for any $\boldsymbol{S}, \boldsymbol{\Sigma}$ positive definite,

$$|||\boldsymbol{S} - \boldsymbol{\Sigma}_0|||_2 < \delta, \quad \textit{and } F(\boldsymbol{S}, \boldsymbol{\Sigma}) < \delta \quad \Longrightarrow \quad |||\boldsymbol{\Sigma} - \boldsymbol{\Sigma}_0|||_2 < \varepsilon .$$

Proof. Fix $\varepsilon > 0$ and write

$$m_0 = \lambda_{\min}(\boldsymbol{\Sigma}_0) > 0, \quad M_0 = \lambda_{\max}(\boldsymbol{\Sigma}_0) .$$

Let $\delta_0 = m_0/2$. If $|||\boldsymbol{S} - \boldsymbol{\Sigma}_0|||_2 < \delta_0$, then by Weyl's inequality (see, for example, Golub and Van Loan 2013, Corollary 8.16)

$$\lambda_{\min}(\boldsymbol{S}) \ge m_0 - \delta_0 = m_0/2, \quad \lambda_{\max}(\boldsymbol{S}) \le M_0 + \delta_0 ,$$

hence $|||\boldsymbol{S}|||_2 \le M_1$ where $M_1 = M_0 + \delta_0$.

Define the scalar function

$$g(t) = t - \log t - 1, \quad t > 0 .$$

Then $g(t) \ge 0$, $g(1) = 0$, and g is strictly decreasing on $(0, 1]$ and strictly increasing on $[1, \infty)$ since $g'(t) = 1 - 1/t$.

Now choose

$$\eta = \min\left\{\frac{1}{2}, \frac{\varepsilon}{4M_1}\right\} \in (0, 1/2], \quad \delta_1 = \min\{g(1-\eta), g(1+\eta)\} > 0 ,$$

and finally set

$$\delta = \min\{\delta_0, \delta_1, \varepsilon/2\} > 0 .$$

Assume $|||\boldsymbol{S} - \boldsymbol{\Sigma}_0|||_2 < \delta$ and $F(\boldsymbol{S}, \boldsymbol{\Sigma}) < \delta$. Since $\delta \le \delta_0$, we have $|||\boldsymbol{S}|||_2 \le M_1$ as above.

Next, let

$$\boldsymbol{B} = \boldsymbol{\Sigma}^{-1/2}\boldsymbol{S}\boldsymbol{\Sigma}^{-1/2} ,$$

which is positive definite. Using similarity invariance of trace and determinant,

$$F(\boldsymbol{S}, \boldsymbol{\Sigma}) = \operatorname{tr}(\boldsymbol{\Sigma}^{-1}\boldsymbol{S}) - \log\det(\boldsymbol{\Sigma}^{-1}\boldsymbol{S}) - p = \operatorname{tr}(\boldsymbol{B}) - \log\det(\boldsymbol{B}) - p .$$

Let $\lambda_1, \ldots, \lambda_p$ be the eigenvalues of $\boldsymbol{B}$. Then

$$F(\boldsymbol{S}, \boldsymbol{\Sigma}) = \sum_{i=1}^{p} \big(\lambda_i - \log \lambda_i - 1\big) = \sum_{i=1}^{p} g(\lambda_i)\,.$$

Because each $g(\lambda_i) \geq 0$ and $\sum_i g(\lambda_i) < \delta \leq \delta_1$, we have $g(\lambda_i) < \delta_1$ for every i. By monotonicity of g on $(0,1]$ and $[1,\infty)$, this forces

$$1 - \eta < \lambda_i < 1 + \eta, \qquad i = 1, \ldots, p.$$

Therefore,

$$(1-\eta)\boldsymbol{I}_p \preceq \boldsymbol{B} \preceq (1+\eta)\boldsymbol{I}_p\,.$$

Multiplying left and right by $\boldsymbol{\Sigma}^{1/2}$ yields

$$(1-\eta)\boldsymbol{\Sigma} \preceq \boldsymbol{S} \preceq (1+\eta)\boldsymbol{\Sigma}\,,$$

since $\boldsymbol{\Sigma}$ is positive definite. Rearranging gives

$$\frac{1}{1+\eta}\boldsymbol{S} \preceq \boldsymbol{\Sigma} \preceq \frac{1}{1-\eta}\boldsymbol{S}\,,$$

and hence

$$-\frac{\eta}{1+\eta}\boldsymbol{S} \preceq \boldsymbol{\Sigma} - \boldsymbol{S} \preceq \frac{\eta}{1-\eta}\boldsymbol{S}\,.$$

Taking spectral norms and using $|||\boldsymbol{S}|||_2 \leq M_1$,

$$|||\boldsymbol{\Sigma} - \boldsymbol{S}|||_2 \leq \frac{\eta}{1-\eta}|||\boldsymbol{S}|||_2 \leq \frac{\eta}{1-\eta}M_1 \leq 2M_1\eta \leq \varepsilon/2\,,$$

where we used $\eta \leq 1/2$ to get $(1-\eta)^{-1} \leq 2$.

Finally,

$$|||\boldsymbol{\Sigma} - \boldsymbol{\Sigma}_0|||_2 \leq |||\boldsymbol{\Sigma} - \boldsymbol{S}|||_2 + |||\boldsymbol{S} - \boldsymbol{\Sigma}_0|||_2 < \varepsilon/2 + \delta \leq \varepsilon/2 + \varepsilon/2 = \varepsilon\,.$$

This proves the claim. □

References

Burg, J. P., D. G. Luenberger, and D. L. Wenger (1982). Estimation of structured covariance matrices. *Proceedings of the IEEE 70*(9), 963–974.

Golub, G. H. and C. F. Van Loan (2013). *Matrix computations.* Johns Hopkins University Press.

Horn, R. A. and C. R. Johnson (2012). *Matrix Analysis.* Cambridge University Press.

Kano, Y. (1983). Consistency of estimators in factor analysis. *Journal of the Japan Statistical Society, Japanese Issue 13*(2), 137–144.

Kano, Y. (1986). Conditions on consistency of estimators in covariance structure model. *Journal of the Japan Statistical Society, Japanese Issue 16*(1), 75–80.

Rudin, W. (1976). *Principles of mathematical analysis* (3rd ed.). McGraw-Hill.
